# Supplementary material for: Labor curves based on cervical dilatation over time and their accuracy and effectiveness: A systematic scoping review
Source: PLoS One. 2024 Mar 22;19(3):e0298046. doi: 10.1371/journal.pone.0298046 (PMC10959354; doi:10.1371/journal.pone.0298046)
Supplement: S1 Table — (PDF) [file pone.0298046.s001.pdf]

S1 Table. Characteristics of included studies.

Author, year, country	Design and study population	Information of interest
Studies describing labor curves		
Ashwal et al., 2020, Israel [1]	Retrospective cohort study, inclusion period 2011-16. 35146 women with spontaneous onset of labor and a singleton, live, full-term fetus with cephalic presentation who achieved 10-cm cervical dilatation were included. Excluded if CS was performed during first stage or without a trial of labor, if cervical dilatation was reported less than twice, if labor duration was >25 hours or if the mother received general or spinal analgesia.	Presents mean labor curve for multiparous and nulliparous women and with and without EDA. Presents time intervals from one cm cervical dilatation to another.
Benmessaoud et al., 2022, France [2]	Retrospective cohort study, inclusion period 2010-2018. 359 with spontaneous onset of labor and a singleton, live, full-term fetus with breech presentation who went through a vaginal delivery, were included. Excluded if home birth.	Presents labor curves for those who delivered a baby in breech presentation, stratified by parity. Presents median time intervals from one cm cervical dilatation to another.
Bhat & Panicker, 2020, India, [3]	Prospective observational study, inclusion period 2017-2018. 1023 women with spontaneous onset of labor and a singleton, live, full-term fetus with cephalic presentation who delivered vaginally, were included. Excluded if malpresentations.	Presents labor curves stratified by parity. Presents median rate of cervical dilatation from 1cm to another from 2-7 cm.
Blankenship et al., 2019, USA [4]	Retrospective cohort study, inclusion period 2004-14. 17097 women with a singleton, full-term fetus with cephalic presentation who achieved 10-cm cervical dilatation were included. Excluded if known fetal congenital anomalies or known contraindications for vaginal delivery.	Presents mean labor curves of those who delivered LGA and AGA infants. They also present labor curves for LGA infants depending on parity, induction vs spontaneous labor onset, obesity and diabetes.
Cahill et al., 2012, USA [5]	Retrospective cohort study, inclusion period 2004-2006. 2373 women with a singleton, full-term fetus with cephalic presentation who achieved 10-cm cervical dilatation and had a umbilical cord gas level obtained at delivery, were included.	Presents median labor curves depending on fetal gender and parity.
Duignan et al., 1975, UK [6]	Prospective cohort study from 1975. 1306 women with spontaneous labor onset, a singleton pregnancy, a fetus with cephalic presentation and who were not given EDA or oxytocic drugs, nor required instrumental or operative delivery were included. Excluded if infant weighed < 2,5kg or if full cervical dilatation at first vaginal examination.	Presents mean labor curves stratified by ethnicity (asian, black and white women) and parity.
Feghali et al., 2015, USA [7]	Retrospective cohort study, inclusion period 2002-2008. Includes 6555 women who underwent medically indicated IOL at < 37 weeks of gestation and a control group of 68965 women who underwent IOL between gestational age 37 and 41+6. Excluded if vaginal delivery was contraindicated due to preexisting conditions and if data from cervical examination on admission were lacking.	Presents mean labor curves for women undergoing preterm IOL, stratified by gestational age and parity.
Ferrazzi et al., 2015, Italy [8]	Prospective observational study, inclusion period 2013. 328 low-risk women with a singleton fetus in cephalic presentation at term were included. Excluded if complicated obstetric history, arising complications in labor, EDA, and failure to progress. See article for detailed criteria	Presents labor curve centiles by parity for the present population and compares them with known median labor curves.

Friedman, 1954, USA [9]	Prospective cohort study.<br>100 women with mainly spontaneous labor onset.	Presents the original "Friedman"-curve: mean labor curve for nulliparous women.
Friedman, 1955, [10]	Cohort study.<br>500 women out of a series of 622 consecutive nulliparous on term were included due to sufficiently detailed, accurate and complete labor charts. Ideal labor curve based on 200 "ideal" cases – meaning no inertia, precipitate labors, OPP, breech, mid-forceps, CS, multiple gestations, heavy mediaction, caudal anasthesia, Pitocin, babies <2,5kg, babies >4kg.	Presents the mean labor curve, the limits of normal and an "ideal" labor curve.
Friedman, 1956, [11]	Cohort study.<br>500 women at term, para 1-5.	Presents mean labor curve for multiparous women and curves stratified by sedation, caudal anesthesia, and occiput posterior position. Also compares curve with that of nulliparous women.
Friedman, 1957, USA [12]	Retrospective cohort study, inclusion period 1952-1956.<br>Includes 236 women at term with cephalopelvic disproportion (CPD) who were eligible for trial of labor.	Presents a labor curve for women diagnosed with CPD prior to labor.
Friedman & Kroll, 1972, USA [13]	Retrospective cohort study, inclusion period 1959-1965.<br>10114 women who had single births, no CS and viable infants and were included, given that the fetal presentation was known of interest for the study (OA, OT, OP, breech).	Presents mean labor curves for women with OA, OP, OT and breech presentations (as determined to exist in the second stage).
Grantz et al., 2015, USA [14]	Retrospective cohort study based on The Consortium on Safe Labor, inclusion period 2002-2008.<br>2892 multiparous women with TOLAC (second delivery) were compared to 56301 nulliparous women. Included if on term, vertex presentation, singleton gestation and either sponataneous or induced labor onset. Excluded if fetal anomalies, antepartum stillbirths, or poor neonatal outcomes and if the labor resulted in uterine rupture.	Presents mean labor curve for women undergoing TOLAC and compared it with nulliparous women. Presents separate curves for induced vs spontaneous onset of labor.
Graseck et al., 2012, USA [15]	Secondary analysis of a retrospective cohort study, inclusion period 2004-2008.<br>2021 women with term, vertex singletons that reached 10cm cervical dilatation were included.<br>Excluded if induced or augmented with cervical ripening agents or oxytocin or if known fetal anomaly.	Presents mean labor curve for women undergoing TOLAC and compared it with non-TOLAC deliveries. Also presents mean time from one cm cervical dilatation to another for the same two groups.
Guedalia et al., 2023, Israel [16]	Multicenter retrospective cohort study, inclusion period 2003-2019.<br>78292 women (of which 10.532 were grand multiparous) with a singleton, live, full-term fetus with cephalic presentation and a trial of labor, were included. Excluded if elective CS.	Presents labor curves stratified by parity; nulliparous, multiparous and grand multiparous (defined as parity of 6+). Presents median time from one cm cervical dilatation to another.
Gurewitsch et al., 2002, Israel & USA [17]	Retrospective cohort study, inclusion period 1990-1995.<br>3177 women were included of which 1095 grand multiparous, 1174 lower-parity multiparous and 908 nulliparous women. Included if spontaneous labor onset, uncomplicated, singleton pregnancy, vertex presentation and at term (week 36-43). Excluded if prior uterine scar, antenatally diagnosed fetal death or major congenital anomaly,	Presents mean labor curves by parity.
Harper et al., 2012, USA [18]	Retrospective cohort study, inclusion period 2004-2008.<br>5388 women at term who reached the second stage of labor, carried a singleton pregnancy in vertex presentation and had an umbilical cord gas obtained at delivery were included. Excluded if congenital anomalies.	Presents average labor curves for induced and spontaneous labor stratified by parity. Also presents median time from one cm cervical

		dilatation to another for spontaneous, induced, and augmented labor.
Hendricks et al., 1970, USA [19]	Prospective cohort study from 1970.<br>303 women included, both induced and spontaneous labor onset and 5 women who had a CS.	Presents mean labor curve for nulliparous and multiparous as well as “normal” curves for those whose labors exhibited no dysfunctional components
Hochler et al., 2021, Israel [20]	Multicenter retrospective cohort study, inclusion period 2003-2017.<br>Includes 1375 twin gestations and 142659 singleton gestations as a control group. The inclusion criteria were a gestational age of 34 weeks and cephalic presentation (of the presenting twin for twin gestations). Excluded if fetal demise.	Presents labor curves stratified by twin/singleton gestation, parity, EDA and onset of labor. Presents time from one cm cervical dilatation to another by parity and singleton vs twin.
Hoffman et al., 2006, USA [21]	Retrospective cohort study, inclusion period 2002-2014.<br>Includes 2671 low-risk multiparous women with an elective induction or spontaneous onset of labor between 37+0 and 40+6 weeks of GA. Excluded if maternal of fetal complications in pregnancy, prior caesarean delivery or if clinically indicated induction.	Presents median duration from one cm cervical dilatation to another according to type of labor onset
Inde et al., 2018, Japan [22]	Retrospective cohort study, inclusion period 2008-2015.<br>3172 women who underwent spontaneous deliveries at term with singleton, cephalic and live neonates of appropriate birthweight and without adverse outcomes.	Presents mean labor curves and time intervals from one cm cervical dilatation to another for the normal population, stratified by parity.
Juhasova et al., 2018, Switzerland [23]	Retrospective cohort study, inclusion period 2007-2014.<br>8378 women with live singleton pregnancies, gestational age 34+0 to 42+5 and a baby in vertex presentation who delivered vaginally were included. Excluded if fetal malformations, placenta previa, critical maternal disease or if data was incomplete.	Presents median labor curves stratified by parity and lists impact factors on cervical dilatation rates.
Juntunen & Kirkinen, 1994, Finland [24]	Retrospective cohort study from 1994.<br>42 women in each group – grand multiparous (≥6 previous deliveries), multiparous (para 2 or 3) and nulliparous. Included women had normal pregnancies and spontaneous deliveries at term. Excluded if breech, multiple pregnancy or oxytocin administration.	Presents mean labor curves for grand multiparous compared with nulliparous and multiparous.
Kominiarek et al., 2011, USA [25]	Retrospective cohort study based on CSL, inclusion period 2002-2008.<br>118978 women at term with a singleton gestation were included. Excluded if stillbirth, breech fetal presentation, unknown BMI on admission and if no documented trial of labor. The first delivery from each patient was selected.	Presents median labor curves and time from one cm cervical dilatation to another stratified by BMI and parity
Laughon et al., 2012, USA [26]	Objective study comparing labor patterns in two retrospective cohort studies: CPP, n = 39.491 delivering in 1959-1966 and the CSL, n= 98.359 delivering in 2002-2008. Only women in spontaneous labor with a singleton gestation were included.	Presents average labor curves for nulliparous, primiparous and secundaparous compared between CPP and CSL.
Ledger, 1969, USA, [27]	Prospective cohort study from 1969.<br>Includes 500 nulliparous and 500 multiparous women primarily from middle and upper social class.	Presents mean labor curves stratified by parity.
Leftwich et al., 2015, USA [28]	Retrospective cohort study based on CSL, inclusion period 2002-2008.<br>146904 women with a singleton fetus > 34 weeks of gestation and with cephalic presentation were included, given that they had 2 or more cervical examinations performed. Excluded if fetal anomaly, missing birth weight data or if patient desired a	Presents mean labor curves depending on birth weight for nulliparous and multiparous women who reached 10cm. Also presents median time from one cm cervical dilatation to another.

	repeat CS and presented in labor. Only the first pregnancy was included if a woman had more than one pregnancy in the database.	
Lekprasert, 1972, Thailand [29]	Prospective cohort study, inclusion period 1972.<br>100 nulliparous and 100 multiparous at term with no more than 2cm cervical dilatation on admission were included. Ideal labor curves were based on 74 nulli and 90 multi (excluded cases with CPD, OPP, breech, operative delivery, small babies and large babies).	Present mean labor curves for "ideal" labor and for the total sample stratified by parity. Compares the findings with those of Friedman.
Liu & Kerr Wilson, 1977, UK [30]	Prospective cohort study from 1977.<br>194 women at term who delivered an infant with birthweight 2600 to 4000 g.	Presents a mean labor curve. The graph also portrays the mean values for different subgroups (spontaneous/induced and nulliparous/multiparous).
Lu et al., 2019, USA [31]	Retrospective cohort study based on CSL, inclusion period 2002-2008.<br>3079 Asian American women with singleton gestation at term, vertex presentation, vaginal delivery and a normal perinatal outcome.	Presents mean labor curves and time from one cm cervical dilatation to another by parity, onset of labor and augmentation.
Lundborg et al., 2020, Sweden [32]	Population-based register cohort study based on the Stockholm-Gotland Obstetric Cohort, inclusion period 2008-2014.<br>85408 women with a term, singleton gestation, spontaneous onset of labor, vertex presentation, vaginal delivery and a normal perinatal outcome.	Presents labor curve percentiles for women by parity. Presents median time from one cm to another. Presents staircase 95th percentile of cumulative duration for each cm, based on the cervical dilatation on admission.
Margolis, 1974, South Africa [33]	Cohort study.<br>887 women with black or Indian ethnicity, with spontaneous labor onset and a normal, unassisted vaginal delivery were included.	Presents mean and median labor curves and their associated 10th and 90th percentile for all groups (Black/Indian and nulliparous/multiparous). Presents both cervix-based and time-based graphs.
McPherson et al., 2014, USA [34]	Retrospective cohort study, inclusion period 2004-2008.<br>5388 women nulliparous women with spontaneous labor onset and a singleton, full-term fetus with cephalic presentation were included. Excluded if fetal anomaly or aneuploidy diagnosed prenatally and if delivered by CS before 10cm dilatation.	Presents mean labor curve for women < 18 years of age and compared it with women 18 years or older. Also presents mean time from one cm cervical dilatation to another for the same two groups.
Meibodi et al., 2017, Iran [35]	Prospective cohort study, inclusion period 2013-2014.<br>1527 women at term with a singleton gestation and vertex presentation were included. Excluded if previous preterm labor, congenital anomalies and IUGR.	Presents labor curves by fetal gender, stratified by parity.
Miller et al., 2019, Israel [36]	Retrospective cohort study, inclusion period 2007-2016.<br>Includes 781 women at term with one previous CS in any previous delivery, singleton gestation, vertex presentation and spontaneous onset of labor.<br>Excluded if fetal malformation or macrosomia or if maternal hypertension or diabetes.	Presents labor curves for women who underwent trial of labor after CS (TOLAC). Presents separate curves for those with and without prior vaginal delivery and by EDA.
Norman et al., 2012, USA [37]	Retrospective cohort study, inclusion period 2004-2008.<br>5204 women with spontaneous labor onset and a singleton, full-term fetus with cephalic presentation who achieved 10cm cervical dilatation were included. Excluded if fetal anomaly or if delivered by CS before 10cm dilatation.	Presents mean labor curves for women according to BMI (categorical) and parity.

Oladapo et al., 2018, USA, China, Japan, Nigeria and Uganda [38]	Systematic review, includes studies from 1986-2016. Reviews 7 studies and thereby labor patterns for 99.971 “low-risk” women with normal perinatal outcomes.	Presents median time intervals from one cm cervical dilatation to another, by parity.
Oladapo et al., 2018, Nigeria and Uganda [39]	Prospective multicenter cohort study, inclusion period 2014-2015. 5606 women with singleton, vertex, term gestation with spontaneous labor onset who presented at ≤ 6 cm of cervical dilatation. Only cases with no adverse birth outcomes were included.	Presents average labor curves stratified by parity and augmentation.
Onishi et al., 2022, USA [40]	Retrospective cohort study, secondary analysis of CSL, inclusion period 2002-2008.<br>110325 women with a term, singleton gestation and vertex presentation were included. Excluded if fetal anomalies or stillbirth, abnormal placentation or any contraindication to vaginal delivery, uterine rupture, 5-minute Apgar score of <7, birth injury, and NICU admission.	Presents labor curves for those with cerclage vs without cerclage and traverse times from one cm to next for the same groups.
Peng et al., 1976, Malaysia [41]	Retrospective and prospective cohort study, inclusion period 1974-1975.<br>644 women who were more than 4 feet 10 inches tall, had a spontaneous vaginal delivery at term of a baby in vertex presentation weighing 2280-4100g were included in the retrospective study. Excluded if operations on the uterus had been performed previously. A prospective study of 50 nulliparous and 50 multiparous was performed to validate the accuracy of the graphs.	Presents labor curves for the Malaysian population by parity. States that the prospective study confirms the validity of the graphs to accurately chart the labor progress.
Petrikovsky et al., 1986, USA [42]	Retrospective cohort study, inclusion period 1978-1981.<br>500 women with consecutive, normal but term grand multiparous labors were included. Grand multiparous is defined as a patient who has at least 5 successive vaginal deliveries. Excluded if multiple gestation, abnormal presentation, induction stimulation of labor or operative delivery.	Presents mean labor curve for the grand multiparous women.
Phillips et al., 1977, USA [43]	Retrospective cohort study, inclusion period 1976.<br>598 women at term with a singleton gestation, a baby in vertex presentation and who required EDA were included.	Presents mean labor curves for women receiving EDA, stratified by parity, oxytocic stimulation and mode of delivery.
Pitchaimuthu & Bhaskaran, 2018, India [44]	Prospective observational study, inclusion period 2014-2015.<br>156 nulliparous women at term with a singleton gestation, spontaneous onset of labor, vertex presentation, est. fetal weight of 2,5-3,5 kg and who delivered by normal vaginal delivery with good maternal and neonatal outcome, were included.<br>Excluded if precipitate labor, IUGR, EDA, malposition or other obstetric/medical complications.	Presents a labor curve and the mean time from one cm cervical dilatation to another.
Rajhvajn et al., 1974, Yugoslavia [45]	A retrospective register study and a prospective study.<br>1005 patients were included in the retrospective and 231 in the prospective study. Inclusion depended on height >150cm, spontaneous vaginal delivery at term, singleton pregnancy, vertex presentation and weight of baby of 2,6-4,4kg.	Presents labor curves based on cumulative frequency tables depending on parity. Starts at 2cm, finishes at delivery.
Schiff et al., 1998, Israel [46]	Cohort study with a comparison group, inclusion period 1984-1996. The study group consisted of 163 women at term with twin gestations who went into spontaneous labor. Included if twin A in vertex position and birth weight of ≥ 2500 g.	Presents labor curves by parity for twin gestations compared with singleton gestations within the same population.

	Excluded if augmented with oxytocin, cervical dilatation of > 6cm on admission, were treated with tocolytic agents during 14 days before delivery or had maternal diabetes, hypertensive disorders or short stature (<150cm). The comparison group (n = 163) included women with singleton gestations who met the same criteria and were matched on parity and maternal age.	
Shalev-Ram et al., 2022, Israel [47]	Retrospective cohort study.<br>572 women (of which 422 attempting TOLAC and 150 nulliparous) at term with spontaneous onset of labor, singleton pregnancies, no previous vaginal delivery and cephalic presentation were included. Excluded if >1 previous CS, known fetal malformation, and maternal hypertension or diabetes. Also excluded if fetal macrosomia (birth weight > 9th centile) or IUGR (<10th centile).	Presents a mean labor curve by median by groups nulliparous and achieving VBAC.
Shenouda et al., 2020, Canada [48]	Retrospective cohort study, inclusion period 2013-2014.<br>526 women at term who presented in active labor with a fetus in vertex position. Included vaginal deliveries and CS on the indication failure to progress. Excluded if unknown pre-pregnancy BMI, age <18 or > 40 years, major congenital anomalies, prior CS, no trial of labor, stillbirth, preeclampsia or eclampsia, placental complications, uterine complications and cord complications.	Presents mean labor curves stratified by parity, BMI and mode of delivery.
Shi et al., 2016, China [49]	A prospective observational study, inclusion period 2013-2014. 1200 women (of which 1091 nulliparous) with spontaneous labor onset and a singleton, full-term fetus with cephalic presentation were included. Excluded if use of analgesia in labor, TOLAC, obesity, or other medical conditions such as hypertension, heart disease etc. Women with GDM included if dietary restrictions only. Excluded if adverse perinatal outcomes, CS during trial of labor or full cervical dilatation on admission.	Presents mean labor curves for women according to cervical dilatation at admission for a dilatation of 1, 2 and 3 cm. Presents time from one cm cervical dilatation to another. Compares numbers with Zhang's and Suzuki's findings.
Shindo et al., 2021, Japan [50]	A retrospective cohort study, inclusion period 2011-2019. 9481 women at term with spontaneous onset, cephalic presentation and a vaginal delivery without uterotonic agents and EDA, were included.	Presents labor curves by parity. Presents time from one cervical dilatation cm to another.
Silver et al., 2000, USA [51]	Retrospective cohort study, inclusion period 1994-1998. Includes 32 triplet pregnancies and compares them with 64 twin pregnancies ang 64 singleton pregnancies. Included women delivering after 23 weeks of GA who reached second stage of labor and had cephalic presentation of the first baby. Twin and singleton cohorts were matched for GA (+/- 1 week), cephalic presentation and EDA use.	Presents mean labor curves and percentiles for triplet, twin and singleton gestations.
Sondgeroth et al., 2015, USA [52]	Retrospective cohort study, inclusion period 2004-2008. Includes 473 women with a vertex-presenting fetus and a previous CS who reached second stage of labor.<br>Excluded if known fetal anomaly.	Presents median labor curves for women with previous CS, stratified by onset of labor (induced/spontaneous). Presents time from one cm cervical dilatation to another.
Spain et al., 2014, USA [53]	Retrospective cohort study, inclusion period 2004-2008.<br>224 preterm (<37 weeks) and 5388 term women included. Included if not given tocolysis or if tocolysis failed. Excluded if known anomalous fetus.	Presents median labor curves for women in preterm labor and compares them with labor at term. Presents median time from one cm cervical dilatation to another.
Steward, 1977, Zambia [54]	Prospective cohort study from 1977.	Presents mean labor curves for women stratified by parity.

	212 women (65 nulliparous, 59, multiparous (1-4) and 88 grand multiparous (>4)) with singleton pregnancies, cephalic presentations and normal labors were included. Normal labor was defined as no induction, oxytocic stimulation, EDA nor instrumental delivery and a baby > 2300 g with an Apgar score of > 5 at 1 minute.	
Suzuki et al., 2010, Japan [55]	Retrospective cohort study.<br>2369 nulliparous women with spontaneous labor onset and a singleton, full-term fetus with cephalic presentation, admitted with <7cm cx dilatation and with labor duration of >3hours from admission were included. Excluded if they had EDA or a CS.	Presents a smoothing spline labor curve. Also presents median time from one cm cervical dilatation to another and compares the numbers with Zhang's.
Timofeev et al., 2012, USA [56]	Retrospective cohort study based on CSL, inclusion period 2002-2008.<br>71282 women at term with a singleton gestation in vertex presentation and spontaneous labor resulting in vaginal birth of a live born neonate were included.<br>Excluded if Apgar scores <7 after 5min, birth injury, known IUGR, congenital anomaly or NICU admission	Presents labor curves and median time from one cm cervical dilatation to another according to preDM, GDM or normal control and stratified by parity. Also presents labor curves for the same groups matched for neonatal birth weight and maternal BMI on admission.
Tuuli et al., 2014, USA [57]	Retrospective cohort study, inclusion period 2004-2008.<br>4845 women with singleton term pregnancies who completed the first stage of labor were included. Excluded if fetal anomalies.	Presents mean labor curves stratified by ethnicity and parity and median time from one cm to another.
Vahratian et al., 2006, USA [58]	Retrospective analysis of data gathered for a prospective cohort study, inclusion period 2002-2004.<br>5589 low-risk women at term with a live born infant were included.	Presents mean time from one cm cervical dilatation to another (from 4 to 10 cm), stratified by parity.
Vahratian et al., 2004, USA [59]	Retrospective analysis of data gathered for a prospective cohort study (Pregnancy, Infection and Nutrition Study), inclusion period 1995-2002.<br>612 nulliparous women with singleton pregnancy, maternal prepregnancy BMI of ≥ 19,8 kg/m^2 and delivery at term were included.	Presents time from one cm cervical dilatation to another by BMI for all included women, and for those with a vaginal delivery.
van Bogaert, 2004, South Africa [60]	Retrospective cohort study from 2004.<br>Includes 1398 multiparous women whose partographs were charted and who had a spontaneous vaginal delivery.	Presents a customized alert and action line for multiparous women
van Bogaert., 2009, [61]	Retrospective observational study from 2009.<br>An audit of 1595 partographs was performed. Inclusion criteria: Spontaneous nulliparous labor at term with a singleton foetus in vertex presentation. Needed to have a completed partograph. Excluded if medical complications in pregnancy.	Presents customized labor curves for a rural South African nulliparous population and the distribution of the rate of cervical dilatation.
Weissman et al., 1990, Israel [62]	Retrospective cohort study.<br>A total of 264 women at term were included, of which 114 had a cervical cerclage.<br>Excluded if CS, oxytocin augmentation, or a cervical siltation >6cm on admission.	Presents mean labor curves ± SD by parity and cervical cerclage.
Woraschk et al., 1978, Germany [63]	Retrospective cohort study from 1978.<br>Includes 448 women of which 219 nulliparous and 219 multiparous. Excluded if risk pregnancy or labor and if stimulated by oxytocin or given analgesics.	Presents labor curves stratified by parity
Zaki et al., 2013, USA [64]	Retrospective cohort study based on CSL, inclusion period 2002-2008.	Presents mean labor curves and median time from one cm cervical

	120442 women with a known maternal age at term with a singleton gestation and a cephalic position were included. Excluded if prior CS or abnormal neonatal outcomes defined as 5-minute Apgar score < 7, congenital anomalies, birth injury and admission at NICU.	dilatation to another stratified by age and parity
Zhang et al., 2010, USA [65]	Retrospective cohort study based on CSL, inclusion period 2002-2008.<br>Includes 62415 women with a term, singleton gestation, spontaneous onset of labor, vertex presentation, vaginal delivery and a normal perinatal outcome.	Presents mean labor curves and time from one cm to another stratified by parity and staircase lines for nulliparous women stratified by cervical dilation at which women were admitted
Zhang et al., 2010, USA [66]	Retrospective cohort study, inclusion period 1959-1965.<br>26838 women who had a singleton term gestation, spontaneous onset of labor, reached 10 cm cervical dilatation, and vertex fetal presentation were included if the 5-minute Apgar score was ≥ 7. Excluded if severe hypertension in pregnancy, cord prolapse and uterine rupture.	Presents mean labor curves and median time from one cm to another stratified by parity.
Zhang et al., 2002, USA [67]	Retrospective cohort study, inclusion period 1992-1996.<br>Includes 1162 nulliparous women with a singleton pregnancy at term, birth weight 2500g-4000g, spontaneous onset of labor, vertex presentation on admission, cx dilatation < 7cm on admission and duration of labor from admission to delivery > 3 hours. CS were excluded.	Presents mean labor curves and median time from one cm cervical dilatation to another.
Zheng et al., 2019, China [68]	Retrospective cohort study, inclusion period 2015-2017.<br>657 women with a history of a lower uterine segment CS, a spontaneous labor onset and a singleton, full-term fetus with cephalic presentation who underwent a successful TOLAC were included. Excluded if reason for previous CS was unclear, if they had oxytocin or analgesia in labor, placental abruption, uterine rupture, neonatal asphyxia, instrumental delivery or a history with vaginal delivery.	Presents mean labor curve for women undergoing successful VBAC and compared two groups; those with previous trial of labor and those with previous elective CS. Also presents mean time from one cm cervical dilatation to another for the same two groups.
Zimmer, 1951, Germany [69]	Retrospective cohort study, inclusion period 1926-1949.<br>113 women with spontaneous birth of a child in vertex presentation with a birth weight of 3000-4000g, no CPD and a maternal age of 20-30 years.	Presents mean labor curves stratified by parity, maternal age and time of rupture of membranes.
Studies describing labor curves and assessing the accuracy of the curves		
Chen & Chu, 1986, Taiwan [70]	Retrospective and prospective cohort study, inclusion period 1982-1983.<br>First aim: to construct normal labor curves: 500 nulliparous women aged 18-29 with spontaneous vaginal delivery who gave birth to a baby in good condition with birth weight >2,5 kg. No EDA or oxytocic agents were used.	Presents mean labor curves for the normal population.
	Second aim: to assess the accuracy of the constructed lines in distinguishing normal labors from those with abnormal outcomes. 143 women that crossed the alert line were included	Presents mode of delivery for two groups; delivered after alert line and before action line (1) and delivered after action line (2).
Daftary et al., 1977, India [71]	Retrospective and prospective cohort study from 1977.<br>First aim: charts of 96 nulliparous women with normal labors was used to create a nomogram. Normal labor defined as anterior vertex presentation, no induction or oxytocic stimulation, no anesthesia, no instrumental or operative assistance and a baby weighing > 2,5 kg in good condition.	Presents mean labor curves for the normal population. Presents alert and action line based on slope of maximum acceleration.

	Second aim: charts of 104 nulliparous women with abnormal labors were used to assess the distribution of interventions according to the nomogram.	Presents mode of delivery and oxytocin augmentation for different groups; delivered before alert line (A), after alert line and before action line (B) and after action line (C).
Philpott & Castle, 1972, Zimbabwe, [72]	A prospective cohort study published in 1972.<br>First aim: The charts for 100 consecutive women described as normal African nulliparous women were used to create the alert line.	Presents labor curves for the Zimbabwean population and introduces the alert line and action line.
	Second aim: Assesses the constructed alert line. 624 nulliparous women with a singleton pregnancy and cervical dilatation of ≥ 3 cm on admission. Excluded if abnormal fetal presentations, placenta previa or eclampsia.	Presents mode of delivery for three groups; delivered before alert line (1), delivered after alert line and before action line (2) or delivered after action line (3).
Studd, 1973, UK [73]	Presents both an observational study (440 women) and a retrospective cohort study (292 women, inclusion period 1972).<br>First aim: The observational study includes Caucasian who had a normal labor and aims to construct the nomogram. Defines normal labor as no induction or oxytocic stimulation, no EDA, spontaneous vaginal delivery, and a baby weighing > 2500g in good condition.	Presents the nomogram – a labor curve for the British population by parity and cm of cervical dilatation on admission.
	Second aim: The retrospective study includes nulliparous women with spontaneous onset of labor and aims to evaluate the nomograms' ability to separate between normal and abnormal labor.	Presents mode of delivery and Apgar score for those left and right of the nomogram.
Studies assessing the accuracy of labor curves		
Bird, 1974, Papua New Guinea [74]	Descriptive study, inclusion period 1973-1974.<br>Assesses the action line of Philpotts partograph.<br>3012 women with a singleton pregnancy, vertex presentation and with no contraindications such as eclampsia, antepartum hemorrhage, induced labor, previous CS or infant birth weight < 1000g were included.	Presents findings on mode of delivery depending on parity and crossing of the action line.
Bolbol-Haghighi et al., 2015, Iran [75]	Cross sectional study, inclusion period 2011.<br>Assesses the alert line in the WHO partograph.<br>140 healthy women with 0-3 former deliveries, a singleton, full-term fetus with cephalic presentation and a maximum dilatation of 3cm upon admission were included. Excluded if known fetal congenital anomalies.	Presents specificity, sensitivity, positive predictive value and negative predictive value of the alert line with neonatal resuscitation as the primary outcome.
Bonet et al., 2019, Mali, Nigeria, Senegal, South Africa, Uganda, Brazil, Equador, India, Indonesia, Malaysia, Thailand & Iran [76]	Systematic review, includes 13 studies from 1992-2018.<br>Assesses the alert and action lines of the WHO partograph.<br>Includes a total of 20471 women. Diverse population, studies with both women at risk and at low risk, were included in the review.	Presents diagnostic test accuracy measures for the alert and the action line separately.

Cardozo et al., 1982, UK [77]	Prospective cohort study from 1982.<br>Assesses cervimetric patterns compared to Studds' labor stencil.<br>684 nulliparous women admitted in spontaneous labor with a gestational age of >34 weeks were included.	Presents the outcomes type of delivery and neonatal outcome for different cervimetric patterns.
Drouin et al., 1979, Cameroon [78]	Retrospective and prospective cohort, inclusion period 1975.<br>Retrospective: 686 women. Prospective: 1045 women.<br>Assesses alert and action line Philpott & Castle's partograph.<br>Patients with a cervical dilatation of >4cm on admission were excluded.	Presents maternal and neonatal outcomes for different groups; delivered before alert line (1), after alert line and before action line (2) and after action line (3). Presents distribution of patients in each group.
Dujardin et al., 1992, Senegal [79]	Prospective observational study, inclusion period 1990.<br>Assesses the WHO partograph alert line and 3-hour action line.<br>Includes 1022 women with completed partograph.	Presents the need for neonatal rescusitation related to crossed/not crossed alert and action line. Results presented as positive predictive value, relative risk, sensitivity and specificity.
Hunter et al., 1983, Canada [80]	Retrospective cohort study, inclusion period 1981-1983.<br>Assesses labor progress based on Hendricks labor curve and 2-hour alert line and 4 hour action line inspired by Philpott & Castle.<br>300 nulliparous women with spontaneous labor onset and a singleton, full-term fetus with cephalic presentation were included. Excluded if they before 3 cm cervical dilatation received oxytocin or were delivered by CS.	Presents neonatal outcomes for different groups; delivered to the left of the nomogram (1) before the 2-hour alert line (2), after alert line and before action line (3) and after action line (4). Presents distribution of oxytocin and mode of delivery in each group.
Khan & Rizvi, 1995, Pakistan [81]	Prospective cohort study, inclusion period 1988-1991.<br>Assesses the WHO partograph and lag times after the alert line.<br>236 women with a previous lower segment CS were included.	Presents specificity and sensitivity of the action line for uterine scar rupture according to different lag times after crossing the alert line
Khan et al., 1996, Pakistan [82]	Prospective cohort study, inclusion period 1988-1991.<br>Assesses the WHO partograph and lag times after the alert line.<br>236 women with a previous lower segment CS were included.	Presents relative risk of uterine rupture for different partographic zones following the alert line.
Lakshmidevi et al., 2012, India [83]	Prospective observational study, study from 2012.<br>Assesses the WHO partograph alert & action line.<br>Included 200 nulliparous women at term with spontaneous labor onset, cephalic presentation, and a singleton pregnancy.<br>Excluded if obstetric risk factors.	Presents mode of delivery and admission to NICU in relation to a labor curve to the left of the alert line, between the lines and right of the action line.
Orji, 2008, Nigeria [84]	A prospective cohort study, inclusion period 2007.<br>Assesses the WHO partograph alert and action lines.<br>463 women at term with singleton gestations, spontaneous labor onset, admitted with a cervical dilatation of >4 cm following a healthy pregnancy were included. Excluded if eclampsia or antepartum hemorrhage.	Presents neonatal outcome and mode of delivery for nulliparous and multiparous stratified by normal active phase, between alert and action line and reached or crossed action line.
Philpott & Castle, 1972, [85]	A prospective cohort study published in 1972. Assesses the constructed action line.<br>624 nulliparous women with a singleton pregnancy and cervical dilatation of ≥ 3 cm on admission. Excluded if abnormal fetal presentations, placenta previa, antepartum hemorrhage or eclampsia.	Presents Apgar score for those delivered before alert line (1), delivered after alert line and before action line (2) or delivered after action line and according to mode of delivery (3A: spontaneous, 3B: vacuum extraction, 3C: CS).
Rani & Laxmi, 2016, India [86]	A prospective observational study, inclusion period 2011-2013.<br>Assesses the WHO partograph alert and action lines.<br>Included 200 women at term with a spontaneous onset of labor and a singleton fetus in a vertex presentation.	Presents fetal and maternal outcome for groups divided by zone in partograph (before alert line, between alert/action line, after action line).

Sanyal et al., 2014., India, [87]	A prospective observational study, inclusion period 2011-2012.<br>Assesses the WHO partograph alert and action lines.<br>500 women at term with a singleton gestation and a fetus in a vertex presentation were included.	Presents mode of delivery in relation to zone in partograph (before alert line, between alert/action line, after action line).
Shah et al., 2016, India, [88]	Prospective cohort study, inclusion period 2012-2013.<br>Assesses the WHO partograph alert and action lines.<br>248 women at term in spontaneous labor, cephalic presentation and a singleton gestation were included. Excluded if previous uterine surgery, CPD, associated complications like Preeclampsia, eclampsia, anemia, premature rupture of membranes, antepartum hemorrhage or medical illness.	Presents mode of delivery, augmentation of labor (yes/no) and neonatal asphyxia (yes/no) for those left of the alert line, between the lines and right of the action line, stratified by parity.
Shakunthala et al., 2022, India [89]	Prospective observational study, inclusion period 2017-2019.<br>100 nulliparous women at term with a singleton gestation, cephalic presentation with vertex as the presenting component. Excluded if CPD, antepartum hemorrhage or high-risk pregnancies.	Presents mode of delivery according to zone in partograph (before alert line, between alert/action line, after action line). Compares distribution of patients in zones with previous studies.
Shinde et al., 2012, India, [90]	Prospective observational study, inclusion period 2010.<br>Assesses the WHO partograph alert and action lines.<br>Includes 100 women (50 nulli- and 50 multiparous) who were admitted for labor at term with vertex presentation, without any obvious risk factors and who were suitable for vaginal delivery on the initial examination.	Presents mode of delivery according to zone in partograph (before alert line, between alert/action line, after action line).
Souza et al., 2018, Nigeria and Uganga [91]	Prospective cohort study, inclusion period 2014-2015.<br>Assesses the WHO partograph alert line.<br>9995 women with singleton pregnancies with a gestational age of > 34 weeks were included. Inclusion criteria were spontaneous onset of labor presenting at cervical dilatation of ≤ 6 cm or undergoing IOL.	Presents diagnostic accuracy of the alert line in identifying severe adverse birth outcomes, both neonatal and maternal. Results presented as likelihood ratios, odds ratios, sensitivity and specificity and receiver operating characteristic-curves.
Studd et al., 1975, UK [92]	A prospective cohort study, inclusion period 1973-1974.<br>Assesses Studds' labor stencil.<br>741 women with spontaneous onset of labor and a cephalic presentation were included.	Presents mode of delivery for women stratified by parity and left, within two hours to the right or two to four hours to the right of the nomogram and augmented by oxytocin or not managed according to protocol.
Thom et al., 1979, UK [93]	Prospective cohort study, inclusion period 1976.<br>Assesses Studds' labor stencil.<br>Includes 1643 women in spontaneous labor with a singleton, cephalic fetus.	Presents fetal outcome and operative delivery rates for groups divided by race, parity and left/right of the action line
Tirkey & Sing, 2022, India [94]	Prospective observational study, inclusion period 2013-2014.<br>Assesses WHO simplified partogram.<br>300 nulliparous women with singleton pregnancies at term (36-42 weeks) carrying a fetus with cephalic presentation in labor. Excluded if fetal anomaly or acute obstetric complications.	Presents frequencies for mode of delivery, NICU admission, induction and augmentation stratified by left or right of the alert line. Also presents duration of 1st and 2nd stage of labor and total duration of labor for the two groups.
van Bogaert., 2006, South Africa [95]	Retrospective cohort study from 2006.<br>Assesses the WHO partograph and lag times after the alert line.<br>Includes 610 women of which 263 had spontaneous vaginal deliveries and 347 had emergency CS.	Presents relative risk of poor neonatal outcome stratified by mode of delivery and partograph result measured as before and after the alert line and time after crossing the alert line.
Studies assessing the effectiveness of labor curves		

Bernitz et al., 2019, Norway [96]	Multicenter, cluster-RCT, inclusion period 2014-2017.<br>Compares Zhang's guideline with the WHO partograph.<br>7277 nulliparous women with spontaneous labor onset and a singleton, full-term fetus with cephalic presentation. -> Robson group 1.	Presents findings on frequency of intrapartum CS as the primary outcome.
Dalbye et al., 2019, Norway [97]	Multicenter, cluster-RCT, inclusion period 2014-2017.<br>Compares Zhang's guideline with the WHO partograph.<br>7277 nulliparous women with spontaneous labor onset and a singleton, full-term fetus with cephalic presentation. -> Robson group 1.	Presents findings on differences in oxytocin augmentation during labor as the primary outcome.
Dalbye et al., 2020, Norway [98]	A secondary analysis of a multicenter, cluster-RCT, inclusion period 2014-2017.<br>Compares Zhang's guideline with the WHO partograph.<br>Included 7277 nulliparous women with spontaneous labor onset and a singleton, full-term fetus with cephalic presentation. -> Robson group 1.	Presents numbers on duration from one cm to another adhering to either Zhang's guideline or the WHO partograph grouped by vaginal delivery and intrapartum CS.
Lavender et al., 1998, England [99]	RCT, inclusion period 1996-1997.<br>Assesses the effectiveness of 2-, 3- or 4-hour action line in the WHO partograph.<br>928 nulliparous women with spontaneous labor onset and a singleton, full-term fetus with cephalic presentation were included. Excluded if diabetes, fetal anomaly, unsatisfactory admission CTG or women requiring high dependency intrapartum care for any other reason.	Presents and compares findings on maternal satisfaction, interventions performed and maternal and neonatal outcomes.
Lavender et al., 2006, England [100]	RCT, inclusion period 1998-2005.<br>Assesses the effectiveness of 2- or 4-hour action line in the WHO partograph.<br>2975 nulliparous women in spontaneous labor onset and a singleton, full-term fetus with cephalic presentation were included. Excluded if significant maternal disease, fetal malformations or requirement of high-dependency intrapartum care.	Presents findings on frequency of CS and maternal satisfaction as the primary outcomes.
Lavender et al., 2018, Australia, Canada, Egypt, India, Mexico, Nigeria, South Africa, UK [101]	Cochrane review, includes randomized, cluster-randomized and quasi-randomized studies that took place between 1985 and 2016.<br>Assess the effectiveness of different partograph designs.<br>11 studies including a total of 9475 women were included in the review. Most studies only included nulliparous women with uncomplicated, low-risk pregnancies in spontaneous labor. One study only included high-risk nulliparous women.	Compares different partograph designs and the use of partograph vs no partograph. For each comparison, risk ratios are presented for the maternal and neonatal outcomes defined by the trial authors.
Lee et al., 2018, Australia [102]	Pilot randomized trial, inclusion period 2015.<br>Compares the 4-hour WHO action line with a stepped dystocia line.<br>Includes 116 low-risk nulliparous women with spontaneous onset of labor at term, a single gestation and cephalic presentation.	Presents relative risk for interventions during labor, PPH, mode of delivery and composite neonatal outcomes.
Lee et al., 2023, Australia [103]	Parallel randomized single blinded trial, inclusion period 2015-2018.<br>Compares the 4-hour WHO action line with a stepped dystocia line.	Presents relative risk for interventions during labor, duration of labor, PPH, mode of delivery, perineal status, and composite neonatal outcomes.

	228 nulliparous women with public insurance, between the age 16-40 years with a singleton pregnancy and a spontaneous onset and labor at term, were included.<br>Excluded if complicated birth, previous fetal death, uterine anomaly, Rh immunization, preexisting diabetes, previous gestational diabetes, severe asthma, substance use, significant psychiatric disorders and BMI at gestation <17 or >35.	
Orhue et al., 2020, Nigeria [104]	RCT, inclusion period 2008-2015.<br>Assesses the effectiveness of 2- or 4-hour action line in the WHO partograph.<br>640 nulliparous women in active labor with intact fetal membranes and a singleton, vertex and term gestation with spontaneous onset of labor were included. Excluded if prepartum hemorrhage, medical disorders, abnormalities, poor fetal growth or macrosomic fetus.	Presents findings on incidence of prolonged labor, delivery mode, number of days hospitalization after delivery, neonatal outcomes, and maternal satisfaction for each group.
Pandey et al., 2022, India [105]	RCT, inclusion period 2021.<br>Compares the WHO Labor Care Guide with the WHO modified partograph.<br>271 women with a singleton gestation at term with a cephalic presentation and spontaneous onset of labor were included.<br>Excluded if medical comorbidity, previous CS, bad obstetrical history or intrapartum EDA.	Presents maternal findings on mode of delivery, PPH, infection, duration of labor, oxytocin augmentation, and Hb and total leucocyte count after labor.<br>Presents neonatal findings such as birthweight, mean Apgar score at 5 min, vital status at birth, NICU admission, days at the NICU, and neonatal condition on discharge.
Rozsa et al. 2022, Norway [106]	A secondary analysis of a multicenter, cluster-RCT, inclusion period 2014-2017.<br>Compares Zhang's guideline with the WHO partograph.<br>Included 3604 nulliparous women with spontaneous labor onset and a singleton, full-term fetus with cephalic presentation. -> Robson group 1.	Presents findings on childbirth experience.
Sinha et al., 2017, India [107]	Prospective comparative study, study from 2017.<br>Assesses the effectiveness of 2- or 4-hour action line in the WHO partograph.<br>Includes 200 nulliparous, aged 19 to 29 at term with singleton live fetus, cephalic presentation, uncomplicated pregnancy and in spontaneous labor.	Presents and compares findings on interventions performed and neonatal outcomes.
World Health Organization, 1994, Indonesia, Malaysia, Thailand [108]	Longitudinal multicenter RCT, inclusion period 1990-1991.<br>Assesses the effectiveness of the WHO partograph, before and after implementation.<br>After collecting baseline data, the partograph was commenced in all labors over 34 weeks' gestation, except when women were admitted with > 8cm cervical dilatation or immediate CS was indicated. 35484 women were included in the study in total.	Presents findings on mode of delivery, duration of labor, oxytocin augmentation, and postpartum sepsis pre- and post-implementation of the partograph.

WHO, World Health Organization; RCT, randomized controlled trial; CSL, the Consortium on Safe Labor; CPP, the Collaborative Perinatal Project; RR, Relative risk; CS, caesarean section; CPD, cephalopelvic disproportion; NICU, neonatal intensive care unit; PPH, postpartum hemorrhage; IOL, induction of labor; IUGR, intrauterine growth restriction; TOLAC, trial of labor after caesarean section; VBAC, vaginal birth after caesarean section; EDA, epidural analgesia; (G)DM, (gravida) diabetes mellitus

References

1. Ashwal E, Livne MY, Benichou JIC, Unger R, Hiersch L, Aviram A, et al. Contemporary patterns of labor in nulliparous and multiparous women. American journal of obstetrics and gynecology. 2020;222(3):267.e1-.e9. doi: https://dx.doi.org/10.1016/j.ajog.2019.09.035. PubMed PMID: 31574290.
2. Benmessaoud I, Jamey M, Monard B, Metz J-P, Bourtembourg-Matras A, Ramanah R, et al. Analysis of spontaneous labor progression of breech presentation at term. PloS one. 2022;17(3):e0262002. doi: https://dx.doi.org/10.1371/journal.pone.0262002.
3. Bhat C, Panicker S. Labor pattern in South Indian population. Journal of SAFOG. 2020;12(6):372-5. doi: https://dx.doi.org/10.5005/jp-journals-10006-1839.
4. Blankenship SA, Woolfolk CL, Raghuraman N, Stout MJ, Macones GA, Cahill AG. First stage of labor progression in women with large-for-gestational age infants. American journal of obstetrics and gynecology. 2019;221(6):640.e1-.e11. doi: https://dx.doi.org/10.1016/j.ajog.2019.06.042. PubMed PMID: 31238039.
5. Cahill AG, Roehl KA, Odibo AO, Zhao Q, Macones GA. Impact of fetal gender on the labor curve. American journal of obstetrics and gynecology. 2012;206(4):335.e1-5. doi: https://dx.doi.org/10.1016/j.ajog.2012.01.021. PubMed PMID: 22306304.
6. Duignan NM, Studd JW, Hughes AO. Characteristics of normal labour in different racial groups. British Journal of Obstetrics and Gynaecology. 1975;82(8):593-601. PubMed PMID: 1180948.
7. Feghali M, Timofeev J, Huang CC, Driggers R, Miodovnik M, Landy HJ, et al. Preterm induction of labor: predictors of vaginal delivery and labor curves. American journal of obstetrics and gynecology. 2015;212(1):91.e1-7. doi: https://dx.doi.org/10.1016/j.ajog.2014.07.035. PubMed PMID: 25068566.
8. Ferrazzi E, Milani S, Cirillo F, Livio S, Piola C, Brusati V, et al. Progression of cervical dilatation in normal human labor is unpredictable. Acta obstetricia et gynecologica Scandinavica. 2015;94(10):1136-44. doi: https://dx.doi.org/10.1111/aogs.12719. PubMed PMID: 26230291.
9. Friedman EA. The graphic analysis of labor. American journal of obstetrics and gynecology. 1954;68:1568-75.
10. Friedman EA. Primigravid labor. A graphicostatistical analysis. Obstetrics and gynecology. 1955;6(6):567-89. PubMed PMID: 280629055.
11. Friedman EA. Labor in multiparas. (A graphico-statistical analysis). Obstetrics and gynecology. 1956;8(6):691-703. PubMed PMID: 280953699.
12. Friedman EA. Trial of labour: Formulation, application, and retrospective clinical evaluation. Obstetrics and gynecology. 1957;10(1):1-9.
13. Friedman EA, Kroll BH. Computer analysis of labor progression. V. Effects of fetal presentation and position. Journal of Reproductive Medicine. 1972;8(3):117-21. PubMed PMID: 4555343.
14. Grantz KL, Gonzalez-Quintero V, Troendle J, Reddy UM, Hinkle SN, Kominiarek MA, et al. Labor patterns in women attempting vaginal birth after cesarean with normal neonatal outcomes. American journal of obstetrics and gynecology. 2015;213(2):226.e1-6. doi: https://dx.doi.org/10.1016/j.ajog.2015.04.033. PubMed PMID: 25935774.
15. Graseck AS, Odibo AO, Tuuli M, Roehl KA, Macones GA, Cahill AG. Normal first stage of labor in women undergoing trial of labor after cesarean delivery. Obstetrics and gynecology. 2012;119(4):732-6. doi: https://dx.doi.org/10.1097/AOG.0b013e31824c096c. PubMed PMID: 22433336.
16. Guedalia J, Lipschuetz M, Walfisch A, Cohen SM, Sheiner E, Samson AO, et al. Partogram of Grandmultiparous Parturients: A Multicenter Cohort Study. Journal of clinical medicine. 2023;12(2). doi: https://dx.doi.org/10.3390/jcm12020592.

17. Gurewitsch ED, Diament P, Fong J, Huang GH, Popovtzer A, Weinstein D, et al. The labor curve of the grand multipara: does progress of labor continue to improve with additional childbearing? American journal of obstetrics and gynecology. 2002;186(6):1331-8. PubMed PMID: 12066118.
18. Harper LM, Caughey AB, Odibo AO, Roehl KA, Zhao Q, Cahill AG. Normal progress of induced labor. Obstetrics & Gynecology. 2012;119(6):1113-8.
19. Hendricks CH, Brenner WE, Kraus G. Normal cervical dilatation pattern in late pregnancy and labor. American journal of obstetrics and gynecology. 1970;106(7):1065-82. PubMed PMID: 5435658.
20. Hochler H, Guedalia J, Lipschuetz M, Walfisch A, Yagel S, Guedalia Friedman E, et al. Normal labor curve in twin gestation. American Journal of Obstetrics & Gynecology. 2021;225(5):546.e1-.e11. doi: 10.1016/j.ajog.2021.07.019. PubMed PMID: 153300458. Language: English. Entry Date: 20211120. Revision Date: 20211202. Publication Type: journal article.
21. Hoffman MK, Vahratian A, Sciscione AC, Troendle JF, Zhang J. Comparison of labor progression between induced and noninduced multiparous women. Obstetrics and gynecology. 2006;107(5):1029-34. PubMed PMID: 16648407.
22. Inde Y, Nakai A, Sekiguchi A, Hayashi M, Takeshita T. Cervical Dilatation Curves of Spontaneous Deliveries in Pregnant Japanese Females. International Journal of Medical Sciences. 2018;15(6):549-56. doi: https://dx.doi.org/10.7150/ijms.23505. PubMed PMID: 29725244.
23. Juhasova J, Kreft M, Zimmermann R, Kimmich N. Impact factors on cervical dilation rates in the first stage of labor. Journal of Perinatal Medicine. 2018;46(1):59-66. doi: https://dx.doi.org/10.1515/jpm-2016-0284. PubMed PMID: 28688227.
24. Juntunen K, Kirkinen P. Partogram of a grand multipara: different descent slope compared with an ordinary parturient. Journal of Perinatal Medicine. 1994;22(3):213-8. PubMed PMID: 7823261.
25. Kominiarek MA, Zhang J, Vanveldhuisen P, Troendle J, Beaver J, Hibbard JU. Contemporary labor patterns: the impact of maternal body mass index. American journal of obstetrics and gynecology. 2011;205(3):244.e1-8. doi: https://dx.doi.org/10.1016/j.ajog.2011.06.014. PubMed PMID: 21798510.
26. Laughon SK, Branch DW, Beaver J, Zhang J. Changes in labor patterns over 50 years. American journal of obstetrics and gynecology. 2012;206(5):419.e1-9. doi: https://dx.doi.org/10.1016/j.ajog.2012.03.003. PubMed PMID: 22542117.
27. Ledger WJ. Monitoring of labor by graphs. Obstetrics & Gynecology. 1969;34(2):174-81.
28. Leftwich HK, Gao W, Wilkins I. Does increase in birth weight change the normal labor curve? American journal of perinatology. 2015;32(1):87-92. doi: https://dx.doi.org/10.1055/s-0034-1376180. PubMed PMID: 24839146.
29. Lekprasert V. Monitoring of labour by graph. Journal of the Medical Association of Thailand. 1972;55(11):647-53. PubMed PMID: 4645350.
30. Liu DTY, Kerr Wilson R. Cervical dilatation in spontaneous and induced labors. British Journal of Clinical Practice. 1977;31(11):177-9. PubMed PMID: 8237994.
31. Lu D, Zhang L, Duan T, Zhang J. Labor patterns in Asian American women with vaginal birth and normal perinatal outcomes. Birth. 2019;46(4):608-15. doi: https://dx.doi.org/10.1111/birt.12445. PubMed PMID: 31297872.
32. Lundborg L, Åberg K, Sandström A, Discacciati A, Tilden EL, Stephansson O, et al. First stage progression in women with spontaneous onset of labor: A large population-based cohort study. PloS one. 2020;15(9):e0239724. doi: https://doi.org/10.1371/journal.pone.0239724.

33. Margolis K. Behaviour of the cervix and the presenting vertex in labour in Blacks and Indians of Natal. South African Medical Journal Suid-Afrikaanse Tydskrif Vir Geneeskunde. 1974;48(18):791-8. PubMed PMID: 4825883.
34. McPherson JA, Tuuli M, Odibo AO, Roehl KA, Zhao Q, Cahill AG. Labor progression in teenage women. American journal of perinatology. 2014;31(9):753-8. doi: https://dx.doi.org/10.1055/s-0033-1359722. PubMed PMID: 24338116.
35. Meibodi MR, Mossayebi E, Najmi Z, Moradi Y. Normal labor curve is affected by fetus gender: A cohort study. Medical Journal of the Islamic Republic of Iran. 2017;31:93. doi: https://dx.doi.org/10.14196/mjiri.31.93. PubMed PMID: 29951394.
36. Miller N, Pelleg M, Hag-Yahia N, Daykan Y, Pasternak Y, Biron-Shental T. Labor progression of women attempting vaginal birth after previous cesarean delivery with or without epidural analgesia. Archives of Gynecology and Obstetrics. 2019;299(1):129-34. doi: https://dx.doi.org/10.1007/s00404-018-4956-5. PubMed PMID: 30386990.
37. Norman SM, Tuuli MG, Odibo AO, Caughey AB, Roehl KA, Cahill AG. The effects of obesity on the first stage of labor. Obstetrics and gynecology. 2012;120(1):130-5. doi: https://dx.doi.org/10.1097/AOG.0b013e318259589c. PubMed PMID: 22914401.
38. Oladapo OT, Diaz V, Bonet M, Abalos E, Thwin SS, Souza H, et al. Cervical dilatation patterns of 'low-risk' women with spontaneous labour and normal perinatal outcomes: a systematic review. BJOG: An International Journal of Obstetrics and Gynaecology. 2018;125(8):944-54. doi: https://dx.doi.org/10.1111/1471-0528.14930. PubMed PMID: 28892266.
39. Oladapo OT, Souza JP, Fawole B, Mugerwa K, Perdona G, Alves D, et al. Progression of the first stage of spontaneous labour: A prospective cohort study in two sub-Saharan African countries. PLoS Medicine / Public Library of Science. 2018;15(1):e1002492. doi: https://dx.doi.org/10.1371/journal.pmed.1002492. PubMed PMID: 29338000.
40. Onishi K, Huang JC, Kawakita T. The effect of cervical cerclage on the labor curve. American Journal of Obstetrics & Gynecology MFM. 2022.
41. Peng WW, Aun LM, Sinnathuray TA, Lin WM. Cervicograms of normal labour in Malaysian women. Medical Journal of Malaysia. 1976;30(4):261-3. PubMed PMID: 979726.
42. Petrikovsky B, Cohen M, Moy F, Tancer ML. Labor patterns in grand multiparas. International Journal of Gynaecology and Obstetrics. 1986;24(6):439-41. PubMed PMID: 20419907.
43. Phillips JC, Hochberg CJ, Petrakis JK, Van Winkle JD. Epidural analgesia and its effects on the "normal" progress of labor. American journal of obstetrics and gynecology. 1977;129(3):316-23. PubMed PMID: 900200.
44. Pitchaimuthu N, Bhaskaran S. Labor Pattern Among Primigravida in Local Population. Journal of Obstetrics and Gynaecology of India. 2018;68(6):482-6. doi: https://dx.doi.org/10.1007/s13224-017-1083-3. PubMed PMID: 30416276.
45. Rajhvajn B, Kurjak A, Latin V, Barsic E. Construction and use of a partograph in the management of labour. Zeitschrift für Geburtshilfe und Perinatologie. 1974;178(1):58-62. PubMed PMID: 4829697.
46. Schiff E, Cohen SB, Dulitzky M, Novikov I, Friedman SA, Mashiach S, et al. Progression of labor in twin versus singleton gestations. American journal of obstetrics and gynecology. 1998;179(5):1181-5. PubMed PMID: 9822497.
47. Shalev-Ram H, Miller N, David L, et al. Spontaneous labor patterns among women attempting vaginal birth after cesarean delivery. Journal of Maternal-Fetal and Neonatal Medicine. 2022;35(25):9325-30.
48. Shenouda C, Wijesooriya A, Toufeili A, Miller MR, Penava D, de Vrijer B. Labour Progression in Obese Women: Are Women With Increased Body Mass Index Having Unnecessary Cesarean Sections? Journal of Obstetrics & Gynaecology Canada: JOGC.

2020;42(3):293-300. doi: https://dx.doi.org/10.1016/j.jogc.2019.04.014. PubMed PMID: 31324481.
49. Shi Q, Tan XQ, Liu XR, Tian XB, Qi HB. Labour patterns in Chinese women in Chongqing. BJOG: An International Journal of Obstetrics and Gynaecology. 2016;123 Suppl 3:57-63. doi: https://dx.doi.org/10.1111/1471-0528.14019. PubMed PMID: 27627599.
50. Shindo R, Aoki S, Misumi T, Nakanishi S, Umazume T, Nagamatsu T, et al. Spontaneous labor curve based on a retrospective multi-center study in Japan. Journal of Obstetrics & Gynaecology Research. 2021;47(12):4263-9. doi: 10.1111/jog.15053. PubMed PMID: 153895618. Language: English. Entry Date: 20211216. Revision Date: 20221201. Publication Type: Article.
51. Silver RK, Haney EI, Grobman WA, MacGregor SN, Casele HL, Neerhof MG. Comparison of active phase labor between triplet, twin, and singleton gestations. Journal of the Society for Gynecologic Investigation. 2000;7(5):297-300. PubMed PMID: 11035282.
52. Sondgeroth KE, Stout MJ, Graseck AS, Roehl KA, Macones GA, Cahill AG. Progress of induced labor in trial of labor after cesarean delivery. American journal of obstetrics and gynecology. 2015;213(3):420.e1-5. doi: https://dx.doi.org/10.1016/j.ajog.2015.05.049. PubMed PMID: 26026920.
53. Spain JE, Tuuli M, Caughey AB, Roehl KA, Zhao Q, Cahill AG. Normal first stage of preterm labor. American journal of perinatology. 2014;31(4):315-20. doi: https://dx.doi.org/10.1055/s-0033-1348951. PubMed PMID: 23775065.
54. Steward P. Introduction of partographic records in a District Hospital in Zambia and development of nomograms of cervical dilatation. Medical Journal of Zambia. 1977;11(4):97-9. PubMed PMID: 919786.
55. Suzuki R, Horiuchi S, Ohtsu H. Evaluation of the labor curve in nulliparous Japanese women. American journal of obstetrics and gynecology. 2010;203(3):226.e1-6. doi: https://dx.doi.org/10.1016/j.ajog.2010.04.014. PubMed PMID: 20494329.
56. Timofeev J, Huang CC, Singh J, Driggers RW, Landy HJ. Spontaneous labor curves in women with pregnancies complicated by diabetes. Journal of Maternal-Fetal & Neonatal Medicine. 2012;25(1):20-6. doi: https://dx.doi.org/10.3109/14767058.2012.626923. PubMed PMID: 21955108.
57. Tuuli MG, Odibo AO, Caughey AB, Roehl K, Macones GA, Cahill AG. Are There Differences in the First Stage of Labor between Black and White Women? American Journal of Perinatology. 2014. doi: http://dx.doi.org/10.1055/s-0034-1382254. PubMed PMID: 53208678.
58. Vahratian A, Hoffman MK, Troendle JF, Zhang J. The impact of parity on course of labor in a contemporary population. Birth. 2006;33(1):12-7. PubMed PMID: 16499527.
59. Vahratian A, Zhang J, Troendle JF, Savitz DA, Siega-Riz AM. Maternal prepregnancy overweight and obesity and the pattern of labor progression in term nulliparous women. Obstetrics and gynecology. 2004;104(5 Pt 1):943-51. PubMed PMID: 15516383.
60. van Bogaert LJ. The multigravid partogram--should it be customised? Journal of Obstetrics and Gynaecology. 2004;24(8):881-5. PubMed PMID: 16147642.
61. van Bogaert LJ. Revising the primigravid partogram: does it make any difference? Archives of Gynecology and Obstetrics. 2009;279(5):643-7. doi: https://dx.doi.org/10.1007/s00404-008-0788-z. PubMed PMID: 18777032.
62. Weissman A, Jakobi P, Zahi S, Zimmer EZ. The effect of cervical cerclage on the course of labor. Obstetrics and gynecology. 1990;76(2):168-71. PubMed PMID: 2371020.
63. Woraschk HJ, Ehinger B, Roepke F. Dynamics of cervix dilatation during labor. [German]. Zentralblatt für Gynäkologie. 1978;100(18):1173-7. PubMed PMID: 8389892.

64. Zaki MN, Hibbard JU, Kominiarek MA. Contemporary labor patterns and maternal age. Obstetrics and gynecology. 2013;122(5):1018-24. doi: https://dx.doi.org/10.1097/AOG.0b013e3182a9c92c. PubMed PMID: 24104787.
65. Zhang J, Landy HJ, Branch DW, Burkman R, Haberman S, Gregory KD, et al. Contemporary patterns of spontaneous labor with normal neonatal outcomes. Obstetrics and gynecology. 2010;116(6):1281-7. doi: https://dx.doi.org/10.1097/AOG.0b013e3181fdef6e. PubMed PMID: 21099592.
66. Zhang J, Troendle J, Mikolajczyk R, Sundaram R, Beaver J, Fraser W. The natural history of the normal first stage of labor. Obstetrics and gynecology. 2010;115(4):705-10. doi: https://dx.doi.org/10.1097/AOG.0b013e3181d55925. PubMed PMID: 20308828.
67. Zhang J, Troendle JF, Yancey MK. Reassessing the labor curve in nulliparous women. American journal of obstetrics and gynecology. 2002;187(4):824-8. PubMed PMID: 12388957.
68. Zheng L, Zhang Q, Liao Q, Chen R, Xu R, Han Q, et al. Labor patterns in Chinese women in Fuzhou attempting vaginal birth after previous cesarean delivery: a retrospective cohort study. Journal of International Medical Research. 2019;47(12):6091-9. doi: https://dx.doi.org/10.1177/0300060519884145. PubMed PMID: 31741410.
69. Zimmer K. The time-degree curve of cervical dilatation in cephalic presentation. [Polyglot]. Archiv für Gynäkologie. 1951;179(5):495-513. PubMed PMID: 81297582.
70. Chen HF, Chu KK. Double-lined nomogram of cervical dilatation in Chinese primigravidas. Acta obstetricia et gynecologica Scandinavica. 1986;65(6):573-5. PubMed PMID: 3799153.
71. Daftary SN, Mhatre PN. Cervicographs in the management of labour in primigravidae. J Obstet Gynecol. 1977;27:687-91.
72. Philpott RH, Castle WM. Cervicographs in the management of labour in primigravidae. I. The alert line for detecting abnormal labour. Journal of Obstetrics and Gynaecology of the British Commonwealth. 1972;79(7):592-8. PubMed PMID: 5043422.
73. Studd J. Partograms and nomograms of cervical dilatation in management of primigravid labour. British Medical Journal. 1973;4(5890):451-5. PubMed PMID: 4758447.
74. Bird GC. Cervicographs in the management of labour in primigravidae and multigravidae. Papua New Guinea Medical Journal. 1974;17(4):324-30. PubMed PMID: 4534251.
75. Bolbol-Haghighi N, Keshavarz M, Delvarianzadeh M, Molzami S. Evaluation of the alert line of partogram in recognizing the need for neonatal resuscitation. Iranian Journal of Nursing and Midwifery Research. 2015;20(5):560-4. doi: https://dx.doi.org/10.4103/1735-9066.164513. PubMed PMID: 26457092.
76. Bonet M, Oladapo OT, Souza JP, Gulmezoglu AM. Diagnostic accuracy of the partograph alert and action lines to predict adverse birth outcomes: a systematic review. BJOG: An International Journal of Obstetrics and Gynaecology. 2019;126(13):1524-33. doi: https://dx.doi.org/10.1111/1471-0528.15884. PubMed PMID: 31334912.
77. Cardozo LD, Gibb DM, Studd JW, Vasant RV, Cooper DJ. Predictive value of cervimetric labour patterns in primigravidae. British Journal of Obstetrics and Gynaecology. 1982;89(1):33-8. PubMed PMID: 7059543.
78. Drouin P, Nasah BT, Nkounawa F. The value of the Partogramme in the management of labor. Obstetrics and gynecology. 1979;53(6):741-5. PubMed PMID: 450344.
79. Dujardin B, De Schampheleire I, Sene H, et al. Value of the alert and action lines on the partogram. Lancet (London, England). 1992;339(8805):1336-8.
80. Hunter DJ, Enkin MW, Sargeant EJ, Wilkinson J, Tugwell P. The outcome of prolonged labor as defined by partography and the use of oxytocin: a descriptive study.

American journal of obstetrics and gynecology. 1983;145(2):189-92. PubMed PMID: 6849352.
81. Khan KS, Rizvi A. The partograph in the management of labor following cesarean section. International Journal of Gynaecology and Obstetrics. 1995;50(2):151-7. PubMed PMID: 7589750.
82. Khan KS, Rizvi A, Rizvi JH. Risk of uterine rupture after the partographic 'alert' line is crossed--an additional dimension in the quest towards safe motherhood in labour following caesarean section. Jpma. 1996;The Journal of the Pakistan Medical Association. 46(6):120-2. PubMed PMID: 127213519.
83. Lakshmidevi M, Malini KV, Shetty VH. Partographic analysis of spontaneous labour at term in primigravida. Journal of Obstetrics and Gynaecology of India. 2012;62(6):635-40. doi: https://dx.doi.org/10.1007/s13224-012-0208-y. PubMed PMID: 24293839.
84. Orji E. Evaluating progress of labor in nulliparas and multiparas using the modified WHO partograph. International Journal of Gynaecology and Obstetrics. 2008;102(3):249-52. doi: https://dx.doi.org/10.1016/j.ijgo.2008.04.024. PubMed PMID: 18603248.
85. Philpott RH, Castle WM. Cervicographs in the management of labour in primigravidae. II. The action line and treatment of abnormal labour. Journal of Obstetrics and Gynaecology of the British Commonwealth. 1972;79(7):599-602. PubMed PMID: 5043423.
86. Rani U, Laxmi B. Effect of partographic monitoring on outcomes for women in spontaneous labour at term. IAIM. 2016;3(7):314-20.
87. Sanyal U, Goswami S, Mukhopadhyay P. The role of partograph in the outcome of spontaneous labor. Nepal Journal of Obstetrics and Gynaecology. 2014;9(1):52-7.
88. Shah N, Maitra N, Pagi S. Evaluating role of parity in progress of labour and its outcome using modified WHO partograph. Int J Reprod Contracept Obstet Gynecol. 2016;5(3):860-3.
89. Shakunthala S, Pilli P, Radhika Ganesh Y, Mobine Ahamad S, Mythri K. To detect abnormal labour at an early stage in primigravida by partogram. European Journal of Molecular and Clinical Medicine. 2022;9(3):5923-32.
90. Shinde KK, Bangal VB, Singh RK. Study of course of labour by modified WHO partograph. IJBAR. 2012;3(5):291-6.
91. Souza JP, Oladapo OT, Fawole B, Mugerwa K, Reis R, Barbosa-Junior F, et al. Cervical dilatation over time is a poor predictor of severe adverse birth outcomes: a diagnostic accuracy study. BJOG: An International Journal of Obstetrics and Gynaecology. 2018;125(8):991-1000. doi: https://dx.doi.org/10.1111/1471-0528.15205. PubMed PMID: 29498187.
92. Studd J, Clegg DR, Sanders RR, Hughes AO. Identification of high risk labours by labour nomogram. British Medical Journal. 1975;2(5970):545-7. PubMed PMID: 1148699.
93. Thom MH, Chan KK, Studd JW. Outcome of normal and dysfunctional labor in different racial groups. American journal of obstetrics and gynecology. 1979;135(4):495-8. PubMed PMID: 484647.
94. Tirkey D, Singh A. Evaluate the Progress of Labour in Primigravida using WHO Simplified Partogram. International Journal of Pharmaceutical and Clinical Research. 2022;14(10):253-63.
95. Van Bogaert LJ. The partogram's result and neonatal outcome. Journal of Obstetrics and Gynaecology. 2006;26(4):321-4. PubMed PMID: 16753681.
96. Bernitz S, Dalbye R, Zhang J, Eggebo TM, Froslie KF, Olsen IC, et al. The frequency of intrapartum caesarean section use with the WHO partograph versus Zhang's guideline in the Labour Progression Study (LaPS): a multicentre, cluster-randomised controlled trial. Lancet (London, England). 2019;393(10169):340-8. doi: https://dx.doi.org/10.1016/S0140-6736(18)31991-3. PubMed PMID: 30581039.

97. Dalbye R, Bernitz S, Olsen CI, et al. The Labor Progression Study (LaPS): The use of oxytocin augmentation during labor following Zhang's guideline and the WHO partograph in a cluster randomized trial. Acta obstetricia et gynecologica Scandinavica. 2019;98(9):1187-94.
98. Dalbye R, Blix E, Froslie KF, Zhang J, Eggebo TM, Olsen IC, et al. The Labour Progression Study (LaPS): Duration of labour following Zhang's guideline and the WHO partograph - A cluster randomised trial. Midwifery. 2020;81:102578. doi: https://dx.doi.org/10.1016/j.midw.2019.102578. PubMed PMID: 31783231.
99. Lavender T, Alfirevic Z, Walkinshaw S. Partogram action line study: a randomised trial. British Journal of Obstetrics and Gynaecology. 1998;105(9):976-80. PubMed PMID: 9763048.
100. Lavender T, Alfirevic Z, Walkinshaw S. Effect of different partogram action lines on birth outcomes: a randomized controlled trial. Obstetrics and gynecology. 2006;108(2):295-302. PubMed PMID: 16880298.
101. Lavender T, Cuthbert A, Smyth RM. Effect of partograph use on outcomes for women in spontaneous labour at term and their babies. Cochrane Database of Systematic Reviews. 2018;8:CD005461. doi: https://dx.doi.org/10.1002/14651858.CD005461.pub5. PubMed PMID: 30080256.
102. Lee NJ, Neal J, Lowe NK, Kildea SV. Comparing Different Partograph Designs for Use in Standard Labor Care: A Pilot Randomized Trial. Maternal & Child Health Journal. 2018;22(3):355-63. doi: https://dx.doi.org/10.1007/s10995-017-2366-0. PubMed PMID: 28936715.
103. Lee N, Flynn J, Gao Y, Kildea S. Comparing compliance with commencement and use of two partograph designs for women in active labour: A randomised controlled trial. Women and birth : journal of the Australian College of Midwives. 2023;36(1):e17-e24. doi: https://dx.doi.org/10.1016/j.wombi.2022.04.004.
104. Orhue A, Oseihie, II, Aziken M, Ande B. Randomized controlled trial of labor outcomes with action line placement at 2 hours versus 4 hours on the partograph. International Journal of Gynaecology and Obstetrics. 2020;150(1):64-71. doi: https://dx.doi.org/10.1002/ijgo.13144. PubMed PMID: 32301113.
105. Pandey D, Bharti R, Dabral A, Khanam Z. Impact of WHO Labor Care Guide on reducing cesarean sections at a tertiary center: an open-label randomized controlled trial. AJOG global reports. 2022;2(3):100075. doi: https://dx.doi.org/10.1016/j.xagr.2022.100075.
106. Rozsa DJ, Dalbye R, Bernitz S, Blix E, Dalen I, Braut GS, et al. The effect of Zhang's guideline vs the WHO partograph on childbirth experience measured by the Childbirth Experience Questionnaire in the Labor Progression Study (LaPS): A cluster randomized trial. Acta obstetricia et gynecologica Scandinavica. 2022;101(2):193-9. doi: https://dx.doi.org/10.1111/aogs.14298.
107. Sinha D, Shrivastava S, Shrivastava S. A comparative study of 4-hour versus 2-hour action line on WHO modified partograph. International Journal of Research in Medical Sciences. 2017;5(3):876-9.
108. World Health Organization. World Health Organization partograph in management of labour. World Health Organization Maternal Health and Safe Motherhood Programme. Lancet (London, England). 1994;343(8910):1399-404. PubMed PMID: 7910888.
