# Supplementary material for: Labor curves based on cervical dilatation over time and their accuracy and effectiveness: A systematic scoping review
Source: PLoS One. 2024 Mar 22;19(3):e0298046. doi: 10.1371/journal.pone.0298046 (PMC10959354; doi:10.1371/journal.pone.0298046)
Supplement: S1 File — (PDF) [file pone.0298046.s002.pdf]

S1 File. Initial literature search in Medline

The search was performed by Toril M. Hestnes, research librarian at Medical library, University of Oslo.

Medline (Ovid)
Date: 10.07.20
Results: 715

----
1 exp Obstetrics/ (22642)
2 exp Parturition/ (17125)
3 exp Delivery, Obstetric/ (79914)
4 exp Labor, Obstetric/ (46044)
5 exp Labor Pain/ (1145)
6 exp Term Birth/ (2954)
7 exp obstetric labor complications/ (68271)
8 exp obstetric labor, premature/ (26292)
9 or/1-8 (185468)
10 (progress* or duration* or course or phase or phases or stage or stages).tw,kw,kf. (3903372)
11 ((birth or births or birthing or childbirth* or parturi* or labor or labour or laboring or labouring or delivery
or deliveries or delivering) adj2 (progress* or duration* or course or phase or phases or stage or stages)).tw,kw,kf.
(11211)
12 (9 and 10) or 11 (25472)
13 exp Cervix Uteri/ (27379)
14 (open or opening or opens or dilat*).tw,kw,kf. (754077)
15 13 and 14 (1612)
16 ((cervic* or cervix) adj3 (open or opening or opens or dilat*)).tw,kw,kf. (4279)
17 15 or 16 (4814)
18 (partogram* or partograph* or cervicogram* or cervicograph* or cervimetry or cervimeter*).tw,kw,kf. (666)
19 (curve or curves or chart or charts or graph or graphs or graphic* or line or lines or scale or scales or rate or
rates or pattern*).tw,kw,kf. (5664136)
20 18 or 19 (5664506)
21 12 and 17 and 20 (727)
22 (Animal Experimentation/ or exp Animals/ or exp Models, Animal/) not Humans/ (4717038)
23 ((veterinar* or animal or animals or rabbit or rabbits or rodent or rodents or rat or rats or mouse or mice or
hamster or hamsters or pig or pigs or piglet or piglets or porcine or pigeon* or horse* or equine or cow or cows or
bovine or goat or goats or sheep or lamb or lambs or monkey or monkeys or murine or ovine or dog or dogs or canine or
cat or cats or feline or dolphin*) not (patient or patients or human or humans)).ti. (2247868)
24 (addresses or autobiography or bibliography or biography or comment or congresses or consensus development
conference or consensus development conference, nih or dataset or dictionary or directory or editorial or festschrift or

historical article or interactive tutorial or interview or lectures or letter or news or newspaper article or personal
narratives or portraits or technical report or twin study or validation studies or video-audio media).ti. (197362)
25 21 not (22 or 23 or 24) (715)
