# Supplementary material for: Labor curves based on cervical dilatation over time and their accuracy and effectiveness: A systematic scoping review
Source: PLoS One. 2024 Mar 22;19(3):e0298046. doi: 10.1371/journal.pone.0298046 (PMC10959354; doi:10.1371/journal.pone.0298046)
Supplement: S2 File — Detailed second search. (PDF) [file pone.0298046.s003.pdf]

S2 File. Literature search history. Detailed second search.

A search was conducted in the following databases:

Database	Results
Medline (Ovid):	5037
Maternity & Infant Care (Ovid):	2682
Embase (Ovid):	6109
Cochrane Library:	122
Epistemonikos:	411
CINAHL (Ebsco):	5899
Scopus:	704
African Index Medicus:	83
Number of references before removal of duplicates:	21047
Number of references after removal of duplicates:	

Searches were conducted the 14th of September 2020 and 12th of October 2020, by Toril M. Hestnes, senior librerian at Medical Library, University of Oslo.

An update search was conducted the 4th of May 2023, with time limitations from 2020 to 2023:

Database	Results
Medline (Ovid):	1003
Maternity & Infant Care (Ovid):	538
Embase (Ovid):	1528
Cochrane Library:	30
Epistemonikos:	84
CINAHL (Ebsco):	1521
Scopus:	245
African Index Medicus:	67
Number of references before removal of duplicates:	5016
Number of references after removal of duplicates:	3084

Medline (Ovid)
Search date: 11.09.20
Results: 5037

1 exp Delivery, Obstetric/ (80412)
2 exp Pregnancy/ (896627)
3 exp Pregnancy Complications/ (427345)
4 exp Parity/ (25070)
5 exp obstetric labor complications/ (68691)
6 exp Perinatal Care/ (10233)
7 (birth or births or birthing or childbirth* or obstetric* or parturi* or intrapart* or parity or parities or partus or pregnan*).tw,kw,kf. (847844)
8 or/1-7 (1269139)
9 (labor or labors or labour or labours or laboring or labouring or delivery or deliveries or delivering).tw,kw,kf. (587691)

UiO : Universitetsbiblioteket
Medisinsk bibliotek

10 8 and 9 (174580)
11 exp Cervix Uteri/ (27464)
12 (cervic* or cervix).tw,kw,kf. (265372)
13 11 or 12 (270011)
14 exp Dilatation/ or exp Dilatation, Pathologic/ (21945)
15 dilat*.tw,kw,kf. (145975)
16 14 or 15 (155305)
17 13 and 16 (6384)
18 (cervi* adj3 (chang* or open* or dynamic* or examinat*)).tw,kw,kf. (6625)
19 (dilat* adj3 (phase* or rate*)).tw,kw,kf. (1154)
20 17 or 18 or 19 (13558)
21 (progression or progress).tw,kw,kf. (772161)
22 ((centimeter* or cm) adj3 (hour or hours)).tw,kw,kf. (369)
23 ((birth or births or birthing or childbirth* or parturi* or intrapart* or parity or parities or partus or labor or labors or labour or labours or laboring or labouring or delivery or deliveries or delivering) adj3 (duration or length or speed or accelerat* or decelerat* or active or phase* or stage* or advance* or prolong* or programmed or course or division*)).tw,kw,kf. (29555)
24 exp Time Factors/ (1189044)
25 or/21-24 (1955751)
26 (partogram* or partograph* or parturograph* or cervicogram* or cervicograph*).tw,kw,kf. (654)
27 (nomogram* or cervimeter* or cervimetr*).tw,kw,kf. (9760)
28 (8 or 9 or 20) and 27 (836)
29 ((WHO or WHO's or World Health Organization*) adj3 guideline*).tw,kw,kf. (7138)
30 (8 or 9 or 20) and 29 (975)
31 ((Friedman* or Zhang* or Philpott*) adj3 (guideline* or curve or curves or chart or charts or graph or graphs or graphic* or graphing or line or lines or scale or scales or pattern* or plot or plots or plotting or slope or slopes)).tw,kw,kf. (109)
32 (curve or curves or chart or charts or graph or graphs or graphic* or graphing or line or lines or scale or scales or pattern* or plot or plots or plotting or slope or slopes or depicted or visual presentation*).tw,kw,kf. (3549033)
33 26 or 28 or 30 or 31 or 32 (3550850)
34 ((partogram* or partograph* or parturograph* or cervicogram* or cervicograph* or action line* or alert line*) adj5 (labor or labors or labour or labours or laboring or labouring)).tw,kw,kf. (196)
35 10 and 20 and 33 (664)
36 10 and 25 and 33 (3594)
37 10 and 20 and 25 (1545)
38 or/34-37 (5037)

Embase (Ovid) Embase Classic+Embase <1947 to 2020 September 10>
Search date: 11.09.20
Results: 6109

1 exp obstetric delivery/ (163392)
2 exp labor complication/ (215476)
3 exp childbirth/ (64835)
4 exp parity/ (39606)
5 exp obstetric procedure/ (489956)
6 (birth or births or birthing or childbirth* or obstetric* or parturi* or intrapart* or parity or parities or partus).tw,kw. (631827)
7 or/1-6 (1062033)
8 (labor or labors or labour or labours or laboring or labouring or delivery or deliveries or delivering).tw,kw. (791739)
9 7 and 8 (215566)

10 exp uterine cervix/ (35376)
11 (cervic* or cervix).tw,kw. (373704)
12 10 or 11 (380189)
13 exp dilatation/ or exp uterine cervix dilatation/ (24505)
14 dilat*.tw,kw. (242861)
15 13 or 14 (244852)
16 12 and 15 (10476)
17 (cervi* adj3 (chang* or open* or dynamic* or examinat*)).tw,kw. (9321)
18 (dilat* adj3 (phase* or rate*)).tw,kw. (1833)
19 16 or 17 or 18 (20709)
20 (progression or progress).tw,kw. (1141069)
21 ((centimeter* or cm) adj3 (hour or hours)).tw,kw. (693)
22 ((birth or births or birthing or childbirth* or parturi* or intrapart* or parity or parities or partus or labor or labors or labour or labours or laboring or labouring or delivery or deliveries or delivering) adj3 (duration or length or speed or accelerat* or decelerat* or active or phase* or stage* or advance* or prolong* or programmed or course or division*)).tw,kw. (41670)
23 exp time factor/ (35020)
24 or/20-23 (1215111)
25 (partogram* or partograph* or parturograph* or cervicogram* or cervicograph*).tw,kw. (919)
26 (nomogram* or cervimeter* or cervimetr*).tw,kw. (15028)
27 (7 or 8 or 19) and 26 (999)
28 ((WHO or WHO's or World Health Organization*) adj2 guideline*).tw,kw. (8244)
29 (7 or 8 or 19) and 28 (819)
30 ((Friedman* or Zhang* or Philpott*) adj3 (guideline* or curve or curves or chart or charts or graph or graphs or graphic* or graphing or line or lines or scale or scales or pattern* or plot or plots or plotting or slope or slopes)).tw,kw. (174)
31 (curve or curves or chart or charts or graph or graphs or graphic* or graphing or line or lines or scale or scales or pattern* or plot or plots or plotting or slope or slopes or depicted or visual presentation*).tw,kw. (4749481)
32 25 or 27 or 29 or 30 or 31 (4751399)
33 ((partogram* or partograph* or parturograph* or cervicogram* or cervicograph* or action line* or alert line*) adj5 (labor or labors or labour or labours or laboring or labouring)).tw,kw. (276)
34 9 and 19 and 32 (1071)
35 9 and 24 and 32 (3981)
36 9 and 19 and 24 (2163)
37 or/33-36 (6109)

CINAHL (Ebscohost)
Search date: 14.09.20
Results: 5899

S1 (MH "Delivery, Obstetric") OR (MH "Childbirth+") OR (MH "Vaginal Birth") OR (MH "Perinatal Care") OR (MH "Intrapartum Care") OR (MH "Pregnancy+") 216,026
S2 (MH "Labor+") OR (MH "Labor Complications") OR (MH "Labor Stage, Second") OR (MH "Labor Stage, First") OR (MH "Labor Stages") OR (MH "Management of Labor") 16,988
S3 TI (birth or births or birthing or childbirth* or obstetric* or parturi* or intrapart* or parity or parities or partus or pregnan*) OR AB (birth or births or birthing or childbirth* or obstetric* or parturi* or intrapart* or parity or parities or partus or pregnan*) 1,931
S4 S1 OR S2 OR S3 313,077
S5 TX (labor or labors or labour or labours or laboring or labouring or delivery or deliveries or delivering) 252,966
S6 S4 AND S5 65,035
S7 (MH "Cervix") 3,344

UiO : Universitetsbiblioteket
Medisinsk bibliotek

S8 TX (cervic* or cervix) 64,686
S9 S7 OR S8 64,686
S10 (MH "Dilatation+") 4,247
S11 (MH "Cervix Dilatation and Effacement") 871
S12 TX dilat* 25,389
S13 S10 OR S11 OR S12 28,490
S14 S9 AND S13 2,162
S15 TX ((cervi* N3 (chang* or open* or dynamic* or examinat*))) 1,931
S16 TX ((dilat* N3 (phase* or rate*))) 287
S17 S14 OR S15 OR S16 4,164
S18 TX progress or progression 173,252
S19 TX (centimeter* OR cm) N3 (hour OR hours) 103
S20 TX (birth or births or birthing or childbirth* or parturi* or intrapart* or parity or parities or partus or labor or labors or labour or labours or laboring or labouring or delivery or deliveries or delivering) N3 (duration or length or speed or accelerat* or decelerat* or active or phase* or stage* or advance* or prolong* or programmed or course or division*) 12,262
S21 (MH "Time Factors") 169,640
S22 S18 OR S19 OR S20 OR S21 345,562
S23 TX partogram* or partograph* or parturograph* or cervicogram* or cervicograph* 254
S24 TX (nomogram* or cervimeter* or cervimetr*) AND (S4 OR S5 OR S17) 238
S25 TX ((WHO or WHO's or World Health Organization* or Friedman* or Zhang* or Philpott*)) N3 (guideline* or curve or curves or chart or charts or graph or graphs or graphic* or graphing or line or lines or scale or scales or pattern* or plot or plots or plotting or slope or slopes)) AND (S4 OR S5 OR S17) 1,068
S26 TX curve or curves or chart or charts or graph or graphs or graphic* or graphing or line or lines or scale or scales or pattern* or plot or plots or plotting or slope or slopes or depicted or visual presentation* 2,322,463
S27 S23 OR S24 OR S25 OR S26 2,322,861
S28 TX (partogram* or partograph* or parturograph* or cervicogram* or cervicograph* or action line* or alert line*) N5 (labor or labors or labour or labours or laboring or labouring) 89
S29 S6 AND S17 AND S27 897
S30 S6 AND S22 AND S27 5,159
S31 S6 AND S17 AND S22 829
S32 S28 OR S29 OR S30 OR S31 5,899

Maternity & Infant Care Database (MIDIRS) <1971 to July 2020>
Search date: 14.09.20
Results: 2682

1 Delivery.de. (1346)
2 Labour.de. (16741)
3 Labour - prolonged.de. (161)
4 Labour - induced.de. (1833)
5 Labour - arrested.de. (69)
6 Labour complications.de. (1658)
7 Labour augmentation.de. (199)
8 Labour duration.de. (450)
9 (Labour stage - first or Labour stage - second).de. (748)
10 Childbirth.de. (2608)
11 Obstetrics.de. (685)
12 Pregnancy.de. (59999)
13 Parity.de. (540)
14 Perinatal care.de. (973)

UiO : Universitetsbiblioteket
Medisinsk bibliotek

15 Intrapartum care.de. (2622)
16 (birth or births or birthing or childbirth* or obstetric* or parturi* or intrapart* or parity or parities or partus or pregnan*).tw. (177801)
17 or/1-16 (181670)
18 (labor or labors or labour or labours or laboring or labouring or delivery or deliveries or delivering).tw. (71022)
19 17 or 18 (189963)
20 Cervix.de. (10)
21 Cervix uteri.de. (217)
22 (cervic* or cervix).tw. (5948)
23 20 or 21 or 22 (5948)
24 Dilatation.de. (4)
25 dilat*.tw. (2766)
26 24 or 25 (2766)
27 23 and 26 (1585)
28 Cervical dilatation.de. (128)
29 Cervical dilation.de. (5)
30 (cervi* adj3 (chang* or open* or dynamic* or examinat*)).tw. (498)
31 (dilat* adj3 (phase* or rate*)).tw. (130)
32 or/27-31 (1946)
33 (progression or progress).tw. (5846)
34 ((centimeter* or cm) adj3 (hour or hours)).tw. (33)
35 ((birth or births or birthing or childbirth* or parturi* or intrapart* or parity or parities or partus or labor or labors or labour or labours or laboring or labouring or delivery or deliveries or delivering) adj3 (duration or length or speed or accelerat* or decelerat* or active or phase* or stage* or advance* or prolong* or programmed or course or division*)).tw. (8194)
36 Time factors.de. (564)
37 or/33-36 (13944)
38 (partogram* or partograph* or parturograph* or cervicogram* or cervicograph*).tw. (300)
39 (nomogram* or cervimeter* or cervimetr*).tw. (381)
40 (19 or 32) and 39 (294)
41 ((WHO or WHO's or World Health Organization*) adj3 (guideline* or curve or curves or chart or charts or graph or graphs or graphic* or graphing or line or lines or scale or scales or pattern* or plot or plots or plotting or slope or slopes)).tw. (522)
42 Labour curves.de. (9)
43 Friedman curve.de. (22)
44 (curve or curves or chart or charts or graph or graphs or graphic* or graphing or line or lines or scale or scales or pattern* or plot or plots or plotting or slope or slopes or depicted or visual presentation*).tw. (30096)
45 38 or 40 or 41 or 42 or 43 or 44 (30732)
46 ((partogram* or partograph* or parturograph* or cervicogram* or cervicograph* or action line* or alert line*) adj5 (labor or labors or labour or labours or laboring or labouring)).tw. (155)
47 19 and 32 and 45 (419)
48 19 and 37 and 45 (1879)
49 19 and 32 and 37 (818)
50 or/46-49 (2682)

Epistemonikos
Search date: 14.09.20
Results: 411

(title:((title:(labor OR labors OR labour OR labours OR laboring OR labouring OR delivery OR deliveries OR delivering) OR abstract:(labor OR labors OR labour OR labours OR laboring OR

UiO : Universitetsbiblioteket
Medisinsk bibliotek

labouring OR delivery OR deliveries OR delivering)) AND (title:(cervic* OR cervix OR dilat* OR progress*) OR abstract:(cervic* OR cervix OR dilat* OR progress* OR duration OR length OR time OR rate* OR speed OR accelerat* OR decelerat* OR active OR phase* OR stage* OR advance* OR prolong* OR programmed OR course OR division)) AND (title:(partogram* OR partograph* OR parturograph* OR cervicogram* OR cervicograph* OR curve OR curves OR chart OR charts OR graph OR graphs OR graphic* OR graphing OR line OR lines OR scale OR scales OR pattern* OR plot OR plots OR plotting OR slope OR slopes OR depicted OR visual presentation) OR abstract:(partogram* OR partograph* OR parturograph* OR cervicogram* OR cervicograph* OR curve OR curves OR chart OR charts OR graph OR graphs OR graphic* OR graphing OR line OR lines OR scale OR scales OR pattern* OR plot OR plots OR plotting OR slope OR slopes OR depicted OR visual presentation))) OR abstract:((title:(labor OR labors OR labour OR labours OR laboring OR labouring OR delivery OR deliveries OR delivering) OR abstract:(labor OR labors OR labour OR labours OR laboring OR labouring OR delivery OR deliveries OR delivering)) AND (title:(cervic* OR cervix OR dilat* OR progress* OR duration OR length OR time OR rate* OR speed OR accelerat* OR decelerat* OR active OR phase* OR stage* OR advance* OR prolong* OR programmed OR course OR division) OR abstract:(cervic* OR cervix OR dilat* OR progress*)) AND (title:(partogram* OR partograph* OR parturograph* OR cervicogram* OR cervicograph* OR curve OR curves OR chart OR charts OR graph OR graphs OR graphic* OR graphing OR line OR lines OR scale OR scales OR pattern* OR plot OR plots OR plotting OR slope OR slopes OR depicted OR visual presentation) OR abstract:(partogram* OR partograph* OR parturograph* OR cervicogram* OR cervicograph* OR curve OR curves OR chart OR charts OR graph OR graphs OR graphic* OR graphing OR line OR lines OR scale OR scales OR pattern* OR plot OR plots OR plotting OR slope OR slopes OR depicted OR visual presentation))))

Cochrane Library
Search date: 14.09.20
Results: 122

#1 MeSH descriptor: [Delivery, Obstetric] explode all trees5167
#2 MeSH descriptor: [Pregnancy] explode all trees 21748
#3 MeSH descriptor: [Pregnancy Complications] explode all trees 11654
#4 MeSH descriptor: [Parity] explode all trees 869
#5 MeSH descriptor: [Obstetric Labor Complications] explode all trees 3874
#6 MeSH descriptor: [Perinatal Care] explode all trees 567
#7 (birth or births or birthing or childbirth* or obstetric* or parturi* or intrapart* or parity or parities or partus or pregnan*):ti,ab,kw (Word variations have been searched) 84273
#8 #1 or #2 or #3 or #4 or #5 or #6 or #7 85331
#9 (labor or labors or labour or labours or laboring or labouring or delivery or deliveries or delivering):ti,ab,kw (Word variations have been searched) 85682
#10 #8 or #9 146350
#11 #8 and #9 24663
#12 MeSH descriptor: [Cervix Uteri] explode all trees 1043
#13 (cervic* or cervix):ti,ab,kw (Word variations have been searched) 21900
#14 #12 or #13 21900
#15 MeSH descriptor: [Dilatation] explode all trees 425
#16 MeSH descriptor: [Dilatation, Pathologic] explode all trees 207
#17 (dilat*):ti,ab,kw (Word variations have been searched) 13097
#18 #15 or #16 or #17 13097
#19 #14 and #18 2216
#20 (cervi* n3 (chang* or open* or dynamic* or examinat*)):ti,ab,kw (Word variations have been searched) 6
#21 (dilat* n3 (phase* or rate*)):ti,ab,kw (Word variations have been searched) 0
#22 #19 or #20 or #21 2222

#23 (progression or progress):ti,ab,kw (Word variations have been searched)92036
#24 ((birth or births or birthing or childbirth* or parturi* or intrapart* or parity or parities or partus or labor or labors or labour or labours or laboring or labouring or delivery or deliveries or delivering) n3 (duration or length or speed or accelerat* or decelerat* or active or phase* or stage* or advance* or prolong* or programmed or course or division*)):ti,ab,kw (Word variations have been searched) 33
#25 MeSH descriptor: [Time Factors] explode all trees 64356
#26 #23 or #24 or #25 152694
#27 (partogram* or partograph* or parturograph* or cervicogram* or cervicograph or curve or curves or chart or charts or graph or graphs or graphic* or graphing or line or lines or scale or scales or pattern* or plot or plots or plotting or slope or slopes or depicted or visual presentation*):ti,ab,kw (Word variations have been searched) 319246
#28 ((WHO or WHO's or World Health Organization or Friedman* or Zhang* or Philpott*) n3 (guideline* or curve or curves or chart or charts or graph or graphs or graphic* or graphing or line or lines or scale or scales or pattern* or plot or plots or plotting or slope or slopes)):ti,ab,kw (Word variations have been searched) 73
#29 #27 or #28 319253
#30 #10 and #22 and #29 543
#31 #10 and #26 and #29 3447
#32 #10 and #22 and #26 327
#33 #30 or #31 or #32 4135 (122 Cochrane reviews)

SCOPUS
Search date: 12.10.20
Results: 704

((((INDEXTERMS ("obstetric delivery") OR INDEXTERMS ("obstetric labor") OR INDEXTERMS ("obstetric labour")) OR ((TITLE-ABS-KEY (birth OR births OR birthing OR childbirth* OR obstetric* OR parturi* OR perinatal OR intrapart* OR parity OR parities OR partus OR pregnan*)) AND (TITLE-ABS-KEY (labor OR labors OR labour OR labours OR laboring OR labouring OR delivery OR deliveries OR delivering)))) AND ((TITLE-ABS-KEY (progress OR progression)) OR (INDEXTERMS ("time factors")) OR (TITLE-ABS ((centimeter* OR cm) PRE/3 (hour OR hours))) OR (TITLE-ABS ((birth OR births OR birthing OR childbirth* OR parturi* OR intrapart* OR parity OR parities OR partus OR labor OR labors OR labour OR labours OR laboring OR labouring OR delivery OR deliveries OR delivering) W/3 (duration OR length OR speed OR accelerat*)))) AND ((TITLE-ABS-KEY (partogram* OR partograph* OR parturograph* OR cervicogram* OR cervicograph*)) OR ((((INDEXTERMS ("obstetric delivery") OR INDEXTERMS ("obstetric labor") OR INDEXTERMS ("obstetric labour")) OR ((TITLE-ABS-KEY (birth OR births OR birthing OR childbirth* OR obstetric* OR parturi* OR perinatal OR intrapart* OR parity OR parities OR partus OR pregnan*)) AND (TITLE-ABS-KEY (labor OR labors OR labour OR labours OR laboring OR labouring OR delivery OR deliveries OR delivering)))) OR (((TITLE-ABS-KEY (dilat*)) AND ((INDEXTERMS ("cervix uteri")) OR (TITLE-ABS-KEY (cervic* OR cervix)))) OR (TITLE-ABS-KEY (cervi* PRE/3 (chang* OR open* OR dynamic* OR examinat*))) OR (TITLE-ABS-KEY (dilat* W/3 (phase* OR rate*))))) AND (TITLE-ABS-KEY (nomogram* OR cervimeter* OR cervimetr*))) OR ((((INDEXTERMS ("obstetric delivery") OR INDEXTERMS ("obstetric labor") OR INDEXTERMS ("obstetric labour")) OR ((TITLE-ABS-KEY (birth OR births OR birthing OR childbirth* OR obstetric* OR parturi* OR perinatal OR intrapart* OR parity OR parities OR partus OR pregnan*)) AND (TITLE-ABS-KEY (labor OR labors OR labour OR labours OR laboring OR labouring OR delivery OR deliveries OR delivering)))) OR (((TITLE-ABS-KEY (dilat*)) AND ((INDEXTERMS ("cervix uteri")) OR (TITLE-ABS-KEY (cervic* OR cervix)))) OR

(TITLE-ABS-KEY (cervi* PRE/3 (chang* OR open* OR dynamic* OR examinat*))) OR (TITLE-ABS-KEY (dilat* W/3 (phase* OR rate*))))) AND (TITLE-ABS-KEY ((who OR who's OR "World Health Organization") W/3 guideline*))) OR (TITLE-ABS ((friedman* OR zhang* OR philpott*) W/3 (guideline* OR curve OR curves OR chart OR charts OR graph OR graphs OR graphic* OR graphing OR line OR lines OR scale OR scales OR pattern* OR plot OR plots OR plotting OR slope OR slopes))) OR (TITLE-ABS-KEY (curve OR curves OR chart OR charts OR graph OR graphs OR graphic* OR graphing OR line OR lines OR scale OR scales OR pattern* OR plot OR plots OR plotting OR slope OR slopes OR depicted OR "visual presentation")))) OR (((INDEXTERMS ("obstetric delivery") OR INDEXTERMS ("obstetric labor") OR INDEXTERMS ("obstetric labour")) OR ((TITLE-ABS-KEY (birth OR births OR birthing OR childbirth* OR obstetric* OR parturi* OR perinatal OR intrapart* OR parity OR parities OR partus OR pregnan*)) AND (TITLE-ABS-KEY (labor OR labors OR labour OR labours OR laboring OR labouring OR delivery OR deliveries OR delivering)))) AND ((TITLE-ABS-KEY (progress OR progression)) OR (INDEXTERMS ("time factors")) OR (TITLE-ABS ((centimeter* OR cm) PRE/3 (hour OR hours))) OR (TITLE-ABS ((birth OR births OR birthing OR childbirth* OR parturi* OR intrapart* OR parity OR parities OR partus OR labor OR labors OR labour OR labours OR laboring OR labouring OR delivery OR deliveries OR delivering) W/3 (duration OR length OR speed OR accelerat*)))) AND (((TITLE-ABS-KEY (dilat*)) AND ((INDEXTERMS ("cervix uteri")) OR (TITLE-ABS-KEY (cervic* OR cervix)))) OR (TITLE-ABS-KEY (cervi* PRE/3 (chang* OR open* OR dynamic* OR examinat*))) OR (TITLE-ABS-KEY (dilat* W/3 (phase* OR rate*)))))) OR (((INDEXTERMS ("obstetric delivery") OR INDEXTERMS ("obstetric labor") OR INDEXTERMS ("obstetric labour")) OR ((TITLE-ABS-KEY (birth OR births OR birthing OR childbirth* OR obstetric* OR parturi* OR perinatal OR intrapart* OR parity OR parities OR partus OR pregnan*)) AND (TITLE-ABS-KEY (labor OR labors OR labour OR labours OR laboring OR labouring OR delivery OR deliveries OR delivering)))) AND (((TITLE-ABS-KEY (dilat*)) AND ((INDEXTERMS ("cervix uteri")) OR (TITLE-ABS-KEY (cervic* OR cervix)))) OR (TITLE-ABS-KEY (cervi* PRE/3 (chang* OR open* OR dynamic* OR examinat*))) OR (TITLE-ABS-KEY (dilat* W/3 (phase* OR rate*))))) AND ((TITLE-ABS-KEY (partogram* OR partograph* OR parturograph* OR cervicogram* OR cervicograph*)) OR ((((INDEXTERMS ("obstetric delivery") OR INDEXTERMS ("obstetric labor") OR INDEXTERMS ("obstetric labour")) OR ((TITLE-ABS-KEY (birth OR births OR birthing OR childbirth* OR obstetric* OR parturi* OR perinatal OR intrapart* OR parity OR parities OR partus OR pregnan*)) AND (TITLE-ABS-KEY (labor OR labors OR labour OR labours OR laboring OR labouring OR delivery OR deliveries OR delivering)))) OR (((TITLE-ABS-KEY (dilat*)) AND ((INDEXTERMS ("cervix uteri")) OR (TITLE-ABS-KEY (cervic* OR cervix)))) OR (TITLE-ABS-KEY (cervi* PRE/3 (chang* OR open* OR dynamic* OR examinat*))) OR (TITLE-ABS-KEY (dilat* W/3 (phase* OR rate*)))))) AND (TITLE-ABS-KEY (nomogram* OR cervimeter* OR cervimetr*))) OR ((((INDEXTERMS ("obstetric delivery") OR INDEXTERMS ("obstetric labor") OR INDEXTERMS ("obstetric labour")) OR ((TITLE-ABS-KEY (birth OR births OR birthing OR childbirth* OR obstetric* OR parturi* OR perinatal OR intrapart* OR parity OR parities OR partus OR pregnan*)) AND (TITLE-ABS-KEY (labor OR labors OR labour OR labours OR laboring OR labouring OR delivery OR deliveries OR delivering)))) OR (((TITLE-ABS-KEY (dilat*)) AND ((INDEXTERMS ("cervix uteri")) OR (TITLE-ABS-KEY (cervic* OR cervix)))) OR (TITLE-ABS-KEY (cervi* PRE/3 (chang* OR open* OR dynamic* OR examinat*))) OR (TITLE-ABS-KEY (dilat* W/3 (phase* OR rate*)))))) AND (TITLE-ABS-KEY ((who OR who's OR "World Health Organization") W/3 guideline*))) OR (TITLE-ABS ((friedman* OR zhang* OR philpott*) W/3 (guideline* OR curve OR curves OR chart OR charts OR graph OR graphs OR graphic* OR graphing OR line OR lines OR scale OR scales OR pattern* OR plot OR plots OR plotting OR slope OR slopes))) OR (TITLE-ABS-KEY (curve OR curves OR chart OR charts OR graph OR graphs OR graphic* OR graphing OR line OR lines OR scale OR scales OR pattern* OR

plot OR plots OR plotting OR slope OR slopes OR depicted OR "visual presentation"))))) OR (TITLE-ABS-KEY ((partogram* OR partograph* OR parturograph* OR cervicogram* OR cervicograph* OR "action line" OR "alert line") W/5 (labor OR labors OR labour OR labours OR laboring OR labouring))) AND NOT INDEX (medline) AND (LIMIT-TO (DOCTYPE , "ar") OR LIMIT-TO (DOCTYPE , "re") OR LIMIT-TO (DOCTYPE , "cp") OR LIMIT-TO (DOCTYPE , "no") OR LIMIT-TO (DOCTYPE , "sh") OR LIMIT-TO (DOCTYPE , "ed") OR LIMIT-TO (DOCTYPE , "er")) AND (LIMIT-TO (SUBJAREA , "MEDI") OR LIMIT-TO (SUBJAREA , "NURS") OR LIMIT-TO (SUBJAREA , "HEAL"))

African Index Medicus
Search date: 12.10.20
Results: 83

Updated search date: 19.05.23
Results: 67

tw:((ti:(labor OR labors OR labour OR labours OR laboring OR labouring OR delivery OR deliveries OR delivering OR birth OR births OR birthing OR childbirth* OR parturi* OR intrapart* OR parity OR parities OR partus)) OR (mh:(labor OR labors OR labour OR labours OR laboring OR labouring OR delivery OR deliveries OR delivering OR birth OR births OR birthing OR childbirth* OR parturi* OR intrapart* OR parity OR parities OR partus))) AND (la:("en")) AND (year_cluster:[2020 TO 2023])

UiO : Universitetsbiblioteket
Medisinsk bibliotek
