# Supplementary material for: Labor curves based on cervical dilatation over time and their accuracy and effectiveness: A systematic scoping review
Source: PLoS One. 2024 Mar 22;19(3):e0298046. doi: 10.1371/journal.pone.0298046 (PMC10959354; doi:10.1371/journal.pone.0298046)
Supplement: S3 File — (PDF) [file pone.0298046.s004.pdf]

# S3 File. Data charting form.

| Covidence number: | |
| --- | --- |
| Country of origin | |
| Type of study (design) | |
| Aim of study | |
| Population size | |
| Population characteristics (parity, risk factors etc) | |
| Methods - copy/paste from abstract | |
| Results - copy/paste from abstract | |
| Definition of active first stage of labor | |
| Outcomes measured (list all the outcomes) copy/paste from the methods section | |
| Other relevant information | |
