# Supplementary material for: Labor curves based on cervical dilatation over time and their accuracy and effectiveness: A systematic scoping review
Source: PLoS One. 2024 Mar 22;19(3):e0298046. doi: 10.1371/journal.pone.0298046 (PMC10959354; doi:10.1371/journal.pone.0298046)
Supplement: S5 File — (PDF) [file pone.0298046.s006.pdf]

S5 File. Study protocol.

Published 09.07.2020 at Open Science Framwork.

A concept map of labor progression lines demonstrating cervical dilatation over time: protocol for a systematic scoping review

Johanne Mamohau Egenberg Huurnink[1,2], Ellen Blix[2], Elisabeth Hals[3], Anne Kaasen[2], Stine Bernitz[2], Tina Lavender[4], Mia Ahlberg[5], Pål Øian[6], Aase Irene Høifødt[1], Andrea Solnes Miltenburg[7], Aase Serine Devold Pay[1,2]

[1]Department of Obstetrics and Gynecology, Oslo University Hospital, Oslo, Norway; [2]Faculty of Health Sciences, Oslo Metropolitan University, Oslo, Norway;[3] Department of Obstetrics and Gynecology, Innlandet Hospital Trust, Lillehammer, Norway; [4]Centre for Global Women's Health, University of Manchester, Manchester, UK; [5] Clinical Epidemiology Division, Department of Medicine, Solna, Karolinska Institutet, Stockholm, Sweden; [6] Department of Obstetrics and Gynecology, University Hospital of North Norway, Tromsø, Norway; [7]Department of Obstetrics and Gynecology, Akershus University Hospital, Akershus, Norway

INTRODUCTION

Since the 1950s, labor progression has been represented with cervicographs, which depict cervical dilatation over time [1, 2]. Despite the long tradition of labor progression monitoring, global consensus on the definitions of active labor onset and of normal and abnormal progression is lacking [3, 4]. Friedman developed the first labor progression line, based on data from a nulliparous American population, which demonstrated that the minimum cervical dilatation slope for active progression in the 95th percentile of normal labor cases was 1.2 cm/h [1, 2]. In 1972, Philpott and Castle [5, 6] adjusted Friedman's model based on data from a Rhodesian population to enable easy distinction of abnormal from normal (the majority of cases) progression. They added an "alert line" representing a modification of the mean progression rate in the slowest 10% of nulliparous cases, delineating 1 cm cervical dilatation/h from a minimum of 3 cm dilatation. The purpose of the alert line was to provide a user-friendly early warning device to identify those

in need of transfer from peripheral to central hospitals [5]. A parallel “action line” was drawn 4 h to the right of the alert line and indicated the need for medical intervention [6].

The cervicograph with the alert and action lines is a key element of the partograph that has been promoted globally by the World Health Organization (WHO) following a large multicenter study conducted in the 1990s, which yielded promising results in terms of improving labor outcomes [7-10]. Modified versions of the WHO partograph have been developed, with the inclusion of action lines at other intervals [11]. In the last decade, however, the validity of the alert and action lines has been called into question [3, 12, 13]. The partograph is a tool for the monitoring of cervical dilatation, the descent of the presenting part of the fetus, maternal and fetal vital parameters, medical treatments administered, and the frequency and strength of contractions [7], but the cervicograph is the dominant component. Lavender and Bernitz [14] recently emphasized that the use of the partograph is a complex intervention requiring an enabling environment and effective implementation in order to reach its full potential. A systematic review of fetal surveillance in low- and middle-income countries showed that the use of the partograph in combination with intermittent auscultation is associated with improved perinatal outcomes in these contexts [15]. A Cochrane review however, demonstrated that the effect of the routine use of partographs is uncertain, with no clear evidence for the superiority of one partograph design over another [11].

Two recent systematic reviews have addressed labor progression lines. Oladapo et al. [12] explored cervical dilatation patterns and found that the threshold of 1 cm/h in the first stage of labor is unrealistically fast for most women. They questioned the universal application of clinical standards assuming linear labor progress, as the existing body of evidence does not support this pattern. Bonet et al. [13] examined the diagnostic accuracy of the alert and action lines; they concluded that existing evidence does not support the use of the 1 cm/h dilatation threshold to identify those at risk of adverse birth outcomes, and that labor duration is not a good indicator of such outcomes.

In 2010, Zhang et al. [16] proposed a contemporary labor progression line based on data from an American population. Their guideline presents a hyperbolic pattern, with active first-stage labor

beginning at 6 cm cervical dilatation for nulliparous and multiparous women with spontaneous labor onset. The guideline for nulliparous women, developed using data from >27,000 cases, was compared with the modified WHO partograph in a multicenter cluster-randomized controlled trial [17]. The study revealed no significant difference in the frequency of intrapartum caesarean section between groups [17]. A prospective cohort study showed that normal labor progress was slow for many women in Nigeria and Uganda [18]. The authors recommended less emphasis on average labor progression lines for decision making, and the replacement of these indicators with individualized tools [18].

Labor progression lines are thus multifaceted and serve different purposes, depending on their intended use and interpretation in local contexts; they can serve as tools for diagnosis, decision making, early screening, and documentation [5, 13, 19]. Although they have played increasingly vital roles in labor wards since the 1950s, most large trials and reviews conducted in recent decades have failed to prove any advantage of their use. Despite this paucity of evidence, labor progression lines continue to be used widely in obstetric and midwifery practice. The coexistence of different labor progression lines with different purposes, and their persistent roles in obstetric and midwifery care worldwide, provide the rationale for the mapping of the relevant evidence. Mapping of the evidence is understood as systematically and comprehensively identifying, organizing, and summarizing the existing knowledge on a given field of interest [20]. The primary aim of this study is to systematically map and describe existing labor progression lines. Secondary aims are to map the evidence for the lines' effectiveness and accuracy and to identify research gaps. A preliminary search of the Medline, Open Science Framework, and Figshare databases for ongoing reviews was conducted on 31 March 2020. A search of Medline for existing scoping reviews was conducted on 1 April 2020. No study protocol or scoping review on labor progression lines was identified.

METHODS

This protocol follows the scoping review protocol development framework of the Joanna Briggs Institute (JBI) [21], an enhancement of the work of Arksey and O'Malley [22] and Levac et al. [23]. To ensure reporting quality, the PRISMA-ScR checklist, which is consistent with the JBI framework, will be applied before the review manuscript is submitted for publication [24].

Research question and objectives

Research question

The research questions are open, as a scoping review allows for broad exploration of the field of evidence [21]. They are: 1) which labor progression lines demonstrating cervical dilatation over time are described in scientific articles? and 2) what evidence exists for the accuracy and effectiveness of labor progression lines?

Objectives

- To identify different labor progression lines/curves/patterns based on cervical dilatation measures:
    - that have been used or are being used as part of obstetric care; and
    - that are presented in articles published in scientific journals, but not necessarily implemented in obstetric care.
- To identify how labor progression lines are described by determining:
    - their described purpose,
    - the definition of active first-stage labor, and
    - the descriptions of normal and abnormal labor progression.
- To map studies evaluating the accuracy and effectiveness of different labor progression lines.
- To map the outcome measures used to assess labor progression defined by the authors of primary quantitative studies.
- To identify gaps in research on labor progression lines.

Inclusion criteria

Use of the population, concept, and context mnemonic is recommended to identify the focus of and eligibility criteria for scoping reviews [21]. Inclusion criteria are developed in alignment with the research questions and objectives [21]. Primary research articles and systematic reviews will be eligible for inclusion in this scoping review. The eligible population will comprise nulli- and

multiparous women and babies with and without risk factors and complications, including cases of spontaneous and induced labor. The concept involves the examination of labor progression lines/scales/curves describing cervical dilatation against time, and the outcomes used to assess the accuracy and effectiveness of labor progression lines. The context will be health care settings in which women in labor receive care from skilled birth attendants (as defined in each setting).

Search strategy

In accordance with the JBI recommendations, we will perform a three-stage literature search [21]. First, a research librarian will conduct an initial search of the Medline and Maternity and Infant Care databases to identify search terms. An analysis of words used in the titles, abstracts, and keywords of relevant publications will follow. Second, the librarian will search the Medline, Embase, Cinahl, Maternity and Infant Care, Scopus, African Index Medicus, Cochrane Central Register of Controlled Trials, Cochrane Library, and Epistemonikos databases using the terms identified in the first step. The results will be downloaded to the EndNote reference management software (version X9; Clarivate Analytics, USA). Third, the full texts of identified studies will be retrieved, and two reviewers will independently search their reference lists to identify additional sources.

The search strategy will be developed iteratively and cohere with the search process. Thus, it will be described in full in the final review manuscript. To improve transparency, the strategy for the initial Medline search is presented as supplementary material (Attachment 1). Before submitting the review manuscript for publication, we will perform a final search of citations of the included publications in Google Scholar to ensure that all relevant articles have been included.

Study selection

Seven review authors (AIH, AK, ASDP, ASM, EB, EH, and JMEH) will cooperate on selecting studies. Prior to considering the eligibility of identified sources, they will perform a pilot test of source selection according to the JBI framework to increase consistency [21]. The reviewers will screen a random sample of 25 titles/abstracts independently according to pre-set criteria, and meet to discuss and clarify the eligibility criteria. Screening and evidence selection will be

managed using the web-based Covidence systematic review software (Veritas Health Innovation Ltd, Australia). Initially, all sources will be imported from EndNote and duplicates will be removed. The reviewers will independently perform the initial selection of evidence by screening titles and abstracts and determining eligibility based on the inclusion criteria, assigning the labels "yes," "maybe," and "no." Each study will be evaluated by two reviewers. The full texts of all sources labeled "yes" or "maybe" by both reviewers will be reviewed. Those labeled "no" by one reviewer will be placed on a "conflict resolution" list, and a third reviewer will determine their eligibility. Those labeled "no" by both reviewers will be placed on an "irrelevant references" list.

In the second assessment, the full texts of the studies will be assessed by two reviewers independently. Sources in languages not understood by the reviewers will be excluded prior to full text retrieval. The reviewers understand Norwegian, Swedish, Danish, English, Dutch, Icelandic, German, and Kiswahili. The full texts of the identified publications will be retrieved and considered for eligibility according to the inclusion criteria. The reviewers will label them "include" or "exclude". In case of discrepancy and failure to reach consensus, a third reviewer will determine eligibility. In accordance with the JBI framework, no formal quality assessment of the scientific articles will be performed; inclusion will depend solely on the eligibility criteria [21].

Data charting

The aim of data extraction is to provide a descriptive summary of the eligible studies and the results that corresponds to the research question [21]. We have developed a data charting form for this purpose (Attachment 2). The following data will be extracted: country of origin, study type and aim, population size and characteristics, methods, results, type and shape of labor progression line, definition of first-stage active labor, accuracy and effectiveness measures, outcomes measured, population on which the labor progression line is based, and other relevant information.

To ensure a common understanding of the forms and the extraction of all data of interest, the reviewers (AIH, AK, ASDP, ASM, EB, EH, and JMEH) will first chart data from three articles independently. The results will be compared and the data charting form aligned to incorporate

relevant findings. Data will be extracted independently, each study assessed by two reviewers. In case of discrepancy, the two reviewers will reach consensus by discussion. Any change made to the charting forms during the iterative data extraction process will be applied to data extraction from all sources, and described in the review manuscript to ensure transparency [21].

RESULTS

The results of the literature search and eligibility assessment will be presented in a PRISMA flow diagram [25]. The research questions will be answered through a descriptive and thematic summary of the results. The data will be presented in tabular form. A manuscript reporting on the review will be submitted for publication as a scientific article to a journal such as *BJOG: An International Journal of Obstetrics & Gynaecology*, *Midwifery*, *PLoS One*, *Acta Obstetricia et Gynecologica Scandinavica*, or *BMC Systematic Reviews*.

CONCLUSION AND DISCUSSION

Systematic scoping reviews are performed to explore and map the extent of existing evidence for a given research question [21]. As such reviews are time consuming and complex, a pre-defined protocol must be used to improve quality and transparency. By identifying the existing evidence for labor progression lines, we may find research gaps.

REFERENCES

1. Friedman, E.A., *The graphic analysis of labor.* American Journal of Obstetrics & Gynecology, 1954. **68**(6): p. 1568-1575.
2. Friedman, E.A., *Primigravid Labor: A graphicostatistical analysis.* 1955. **6**(6): p. 567-589.
3. World Health Organization. *WHO recommendations. Intrapartum care for a positive childbirth experience*. 2018 [cited 2019 26 April]; Available from: https://extranet.who.int/rhl/topics/preconception-pregnancy-childbirth-and-postpartum-care/care-during-childbirth/care-during-labour-1st-stage/who-recommendation-definitions-latent-and-active-first-stages-labour-0.
4. Abalos, E., et al., *Duration of spontaneous labour in 'low-risk' women with 'normal' perinatal outcomes: A systematic review.* European journal of obstetrics, gynecology, and reproductive biology, 2018. **223**: p. 123-132.
5. Philpott, R. and W. Castle, *Cervicographs in the management of labour in primigravidae: I. The alert line for detecting abnormal labour.* 1972. **79**(7): p. 592-598.
6. Philpott, R. and W. Castle, *Cervicographs in the management of labour in primigravidae: II. The action line and treatment of abnormal labour.* 1972. **79**(7): p. 599-602.
7. World Health Organization, *The Partograph : the application of the WHO partograph in the management of labour, report of a WHO multicentre study, 1990-1991*. 1994, World Health Organization: Geneva.
8. World Health Organization. *WHO recommendations for augmentation of labour*. 2014 [cited 2019 25 April]; Available from: https://www.who.int/reproductivehealth/publications/maternal_perinatal_health/augmentation-labour/en/.
9. World Health Organization. *Managing complications in pregnancy and childbirth: a guide for midwives and doctors*. 2017 [cited 2019 27 April]; 2:[Available from: https://www.who.int/maternal_child_adolescent/documents/managing-complications-pregnancy-childbirth/en/.
10. World Health Organization, *World Health Organization partograph in management of labour.* Lancet, 1994. **343**(8910): p. 1399-404.
11. Lavender, T., A. Cuthbert, and R.M.J.C.D.o.S.R. Smyth, *Effect of partograph use on outcomes for women in spontaneous labour at term and their babies.* 2018(8).
12. Oladapo, O.T., et al., *Cervical dilatation patterns of 'low-risk'women with spontaneous labour and normal perinatal outcomes: a systematic review.* 2018. **125**(8): p. 944-954.
13. Bonet, M., et al., *Diagnostic accuracy of the partograph alert and action lines to predict adverse birth outcomes: a systematic review.* 2019. **126**(13): p. 1524-1533.
14. Lavender, T. and S. Bernitz *Use of the partograph-current thinking*. 2020. DOI: https://doi.org/10.1016/j.bpobgyn.2020.03.010.
15. Housseine, N., et al., *Strategies for intrapartum foetal surveillance in low-and middle-income countries: A systematic review.* 2018. **13**(10).
16. Zhang, J., et al., *Contemporary Patterns of Spontaneous Labor With Normal Neonatal Outcomes.* Obstetrics & Gynecology, 2010. **116**(6): p. 1281-1287.
17. Bernitz, S., et al., *The frequency of intrapartum caesarean section use with the WHO partograph versus Zhang's guideline in the Labour Progression Study (LaPS): a multicentre, cluster-randomised controlled trial.* The Lancet, 2019. **393**(10169): p. 340-348.
18. Oladapo, O.T., et al., *Progression of the first stage of spontaneous labour: a prospective cohort study in two sub-Saharan African countries.* PLoS medicine, 2018. **15**(1): p. e1002492.

19. Miltenburg, A.S., et al., *Quality of care during childbirth in Tanzania: identification of areas that need improvement.* Reproductive Health, 2018. **15**(1): p. 14.
20. Miake-Lye, I.M., et al., *What is an evidence map? A systematic review of published evidence maps and their definitions, methods, and products.* 2016. **5**(1): p. 28.
21. Peters, M., et al., *Chapter 11: Scoping Reviews (2020 version)*, E. Aromataris and Z. Munn, Editors. 2020: Joanna Briggs Institute Reviewer's Manual, JBI, Available from https://reviewersmanual.joannabriggs.org/
22. Arksey, H. and L.J.I.j.o.s.r.m. O'Malley, *Scoping studies: towards a methodological framework.* 2005. **8**(1): p. 19-32.
23. Levac, D., H. Colquhoun, and K.K.J.I.s. O'Brien, *Scoping studies: advancing the methodology.* 2010. **5**(1): p. 69.
24. Tricco, A.C., et al., *PRISMA extension for scoping reviews (PRISMA-ScR): checklist and explanation.* 2018. **169**(7): p. 467-473.
25. Moher, D., et al., *Preferred Reporting Items for Systematic Reviews and Meta-Analyses: The PRISMA Statement.* PLOS Medicine, 2009. **6**(7): p. e1000097.
