# Supplementary material for: Labor curves based on cervical dilatation over time and their accuracy and effectiveness: A systematic scoping review
Source: PLoS One. 2024 Mar 22;19(3):e0298046. doi: 10.1371/journal.pone.0298046 (PMC10959354; doi:10.1371/journal.pone.0298046)
Supplement: Attachment — Submitted filename: Response to reviewers.docx [file pone.0298046.s007.docx]

**Response to the Reviewers**

Dear Editor and Reviewers,

Thank you for providing us with feedback and comments on the manuscript "Labor curves based on cervical dilatation over time and their accuracy and effectiveness: a systematic scoping review". We highly appreciate your contributions; it has helped us enhance our manuscript. Below, you will find our concise point-by-point response to the recommendations provided by reviewer #1. We sincerely hope that you will find the revised version suitable for publication.

Review #1:

1. *Explicitly the exclusion criteria in the summary*
   a. We understand this as “explicitly state the exclusion criteria in the summary”. Exclusion criteria have been added to the abstract.
2. *Specify the methods used to assess the risk of bias*
   a. According to Peters et al. [1], the scoping review methodology does not require assessment of risk of bias. This is due to the aim of scoping reviews of providing an overview of the field, regardless of study quality and potential bias. It can be relevant to conduct risk of bias assessment if a meta-analysis is conducted as part of a scoping review, however this is not typical. We did not conduct a meta-analysis as part of our scoping review and have therefore not assessed the risk of bias in the included studies.
3. *Standardize the study objectives in the summary and in the introduction of your paper*
   a. The study objectives have been standardized accordingly in the summary and in the introduction.
4. *Present risk of bias assessments for each included study*
   a. Please see response to question 2.
5. *Explain in detail methods for assessing confidence in the body of evidence for the outcomes analyzed.*
   a. Methods for assessing confidence in the body of evidence for the outcomes analyzed is understood as linked to critical appraisal of the evidence. According to Peters et al. [1], critical appraisal of the evidence is not applicable for a scoping review. The confidence in the body of evidence in this scoping review, lies in the transparency and the systematic and comprehensive conduct and report of the review.

Due to the inherent nature of a scoping review, implications for practice are rarely made [1]. However, a scoping review can serve as a valuable tool to provide a comprehensive overview of the existing literature, highlighting what has been studied and published within the field of labor curves and identifying areas that require further investigation. The scoping review also includes systematic reviews where the included studies are assessed according to risk of bias. Our review can be seen as a complement to these meta-analyses, and provides a broad picture of the existing evidence, independent of heterogeneity and quality of studies.

Thank you once again for your valuable feedback. We sincerely hope that our revised manuscript complemented by our response to your suggestions, meet your expectations. We look forward to hearing from you soon.

On behalf of the authors,
Johanne M.E. Huurnink

References

1.	Peters M, Godfrey C, McInerney P, Munn Z, Tricco A, Khalil H. Chapter 11: Scoping Reviews (2020 version). 2020. Joanna Briggs Institute Reviewer's Manual, JBI, Available from <https://reviewersmanual.joannabriggs.org/>. Available from: <https://reviewersmanual.joannabriggs.org/>
